# Metabolic Profiling of Sugars and Organic Acids, and Expression Analyses of Metabolism-Associated Genes in Two Yellow-Peel Pitaya Species

**DOI:** 10.3390/plants11050694

**Published:** 2022-03-04

**Authors:** Fangfang Xie, Canbin Chen, Jiaxuan Chen, Yuanju Yuan, Qingzhu Hua, Zhike Zhang, Jietang Zhao, Guibing Hu, Jianye Chen, Yonghua Qin

**Affiliations:** 1 Guangdong Provincial Key Laboratory of Postharvest Science of Fruits and Vegetables, College of Horticulture, South China Agricultural University, Guangzhou 510642, China; xiefangfang202012@163.com (F.X.); poloky2@163.com (Z.Z.); zhaojietang@gmail.com (J.Z.); guibing@scau.edu.cn (G.H.); 2 Key Laboratory of Biology and Genetic Improvement of Horticultural Crops (South China), Ministry of Agriculture and Rural Affairs, College of Horticulture, South China Agricultural University, Guangzhou 510642, China; nnchencanbin@163.com (C.C.); jxchen0127@163.com (J.C.); yyj974028200@163.com (Y.Y.); huaqingzhu@stu.scau.edu.cn (Q.H.)

**Keywords:** yellow-peel pitaya, sugar and organic acid metabolism, transcriptome analyses, gene expression

## Abstract

Sugar and organic acids are important factors determining pitaya fruit quality. However, changes in sugars and acids, and expressions of metabolism-associated genes during fruit maturation of yellow-peel pitayas are not well-documented. In this study, metabolic and expression analyses in pulps of different fruit developmental stages of ‘Wucihuanglong’ (‘WCHL’, *Hylocereus undatus*) and ‘Youcihuanglong’ pitaya (‘YCHL’, *Hylocereus megalanthus*) were used to explore the sugar and organic acid metabolic process. Total phenols and flavonoids were mainly accumulated at S1 in pitaya pulps. Ascorbic acid contents of ‘WCHL’ pitaya were higher than that of ‘YCHL’ pitaya during fruit maturation. Starch was mainly accumulated at early fruit development stages while soluble sugars were rich in late stages. Sucrose, fructose, and glucose were the main sugar components of ‘YCHL’ pitaya while glucose was dominant in ‘WCHL’ pitaya. Malic and citric acids were the main organic acids in ‘WCHL’ and ‘YCHL’ pitayas, respectively. Based on the transcriptome analyses, 118 genes involved in pitaya sugar and organic acid metabolism were obtained. Results from the correlation analyses between the expression profiling of candidate genes and the contents of sugar and organic acid showed that 51 genes had a significant correlation relationship and probably perform key role in pitaya sugar and organic acid metabolism processes. The finding of the present study provides new information for quality regulation of pitayas.

## 1. Introduction

Pitaya (also known as dragon fruit) belonging to *Hylocereus* genus within the Cactaceae family (Caryophyllales order) is native to Mexico, and Central and South America. Nowadays, pitayas are widely commercially cultivated in the tropical and subtropical regions. Based on the color of peel and pulp, pitaya is mainly classified into three species, i.e., *Hylocereus undatus* (*H. undatus*, red or yellow peel with scales and white pulp), *H. monacanthus* or *H. polyrhizus* (red peel with scales and red pulp) and *H. megalanthus* or *Selenicereus megalanthus* (yellow peel without scales and with white pulp) [1]. Pitaya is popular with consumers due to its abundant betalains, exotic appearance, fresh and sweet taste, and high nutrients [2,3,4]. Moreover, yellow-peel pitayas have economic potential in the market due to its conspicuous appearance, shocking yellow color and long shelf life. Pitaya cultivars from a different genetic background have different biochemical and nutritional characteristics. In general, sugar and organic acid metabolism processes are developed during fruit development and maturation with sugar accumulation and organic acid degradation. Besides, sugar and organic acids are crucial components of pitaya fruit quality, including taste, flavor, and pH, which influences the needs of consumers.

During fruit development and ripening, several metabolite processes are presented, of which sugar and organic metabolism perform important roles in fruit quality formation. Starch, as the predominant storage carbohydrate in plants, mainly consists of linear amylose and branched amylopectin. Starch is an important index associated with fruit texture, for instance, the softness of guava [5], and smoothness and dry constancy of pumpkin [6]. Starch synthesis and degradation pathway are involved in the regulation of fruit quality [7,8]. Phosphoglucomutase (PGM), ADP-glucose pyrophosphorylase (AGPS) and starch synthase (StSy) are key regulatory enzymes responsible for starch biosynthesis [8,9,10]. During starch metabolism, the phosphorylation of amylopectin is an essential step which requires close collaboration of dikinases, the glucan, water dikinase (GWD), and phosphoglucan, water dikinase (PWD) [7]. Simultaneously, starch is degraded while the sugars are accumulated during fruit development and ripening [3,6].

The levels of soluble sugars such as glucose, fructose, and sucrose are responsible for fruit sweetness. In citrus, sucrose, fructose, and glucose are the three major carbohydrates which is the main components of total soluble solids [11]. Glucose and fructose are the most abundant molecules in pomegranate [12] and watermelon [13]. Sucrose is the main component of soluble sugars in apricot [14] compared with pear, which is fructose-dominant [15], while red-peel pitaya is glucose-dominant [3,16]. Sucrose phosphate synthase (SPS) is the key enzyme responsible for sucrose synthesis while invertase (Ivr) and sucrose synthase (SuSy) are the main enzymes involved in sucrose catabolism [17,18]. Moreover, only hexokinases (HXKs) and fructokinases (FRKs) are capable of catalyzing the essential irreversible phosphorylation of glucose and fructose in plants [19]. Glucose-6-phosphate isomerase (PGI) catalyzes the interconversion between D-glucose-6-phosphate and D-fructose-6-phosphate which performs important roles in glycolysis and gluconeogenesis [20].

The fruit sourness is principally determined by organic acids (mainly citric and malic acids). Citric acid and malic acid are the main organic acids in pomegranates [12]. Malic acid is dominant in red-peel pitaya [3], watermelon [13], and apple [21] while citric acid is dominant in citrus [11] and pear [22]. The glycolytic pathway is the oxidization of glucose to pyruvate, of which phosphoglycerate kinase (PGK), enolase (Eno), and pyruvate kinase (PK) are key enzymes for catalyzing 1,3-bisphosphoglycerate (1,3-BPGA) to produce pyruvate and ATP [23]. The cofactor acetyl coenzyme A, generated from pyruvate by pyruvate dehydrogenase (PDH), is required directly for the tricarboxylic acid (TCA) cycle and other biochemical reactions [24]. Citrate synthase (CS), aconitate hydratase (ACO), succinyl-CoA synthetase (SCS), 2-oxoglutarate dehydrogenase (OGDH), dihydrolipoyllysine-residue succinyltransferase (DLST), succinate dehydrogenase (SDH), fumarate hydratase (FUM), and malic dehydrogenase (MDH) are also involved in the TCA cycle [25,26].

Ascorbic acid (also named ascorbate or vitamin C) is one of the ubiquitous water-soluble low molecular weight antioxidants in fruits, such as guava [5] and kiwifruit [27]. Ascorbic acid is essential for human health, intaking from edible plants due to humans lack of the last step ascorbate synthetic enzyme [28]. Four de novo biosynthesis pathways of ascorbic acid: the L-galactose, L-gulose, myo-inositol, and D-galacturonate pathways have been proposed in plants [29]. The L-galactose pathway of ascorbate biosynthesis starting from glucose has been extensively studied in higher plants [30]. Mannose-6-phosphate isomerase (PMI), phosphomannomutase (PMM), mannose-1-phosphate guanylyltransferase (GMP), GDP-mannose 3,5-epimerase (GME), GDP-L-galactose phosphorylase (GGP), L-galactose-1-phosphate phosphatase (GPP), L-galactose dehydrogenase (GDH), and L-galactono-1,4-lactone dehydrogenase (GLDH) related to the ascorbate biosynthesis while L-ascorbate oxidase (AO), L-ascorbate peroxidase (APX), dehydroascorbate reductase (DHAR), and monodehydroascorbate reductase (MDAR) control the regeneration of ascorbate [5,30].

Elucidation of key metabolites and candidate genes responsible for fruit quality is beneficial for breeding new pitaya cultivars and improving their nutritional values. To date, candidate genes related to betalain biosynthesis were isolated from transcriptome data of red-peel and yellow-peel pitayas [31,32]. Besides, several candidate genes involved in sugar biosynthesis were also obtained through RNA-Seq [16]. However, compared with red-peel pitayas, genes related to sugars and organic acids in fruit development and maturation of yellow-peel pitaya are rarely studied. In this study, nine fruit development stages of ‘WCHL’ and ‘YCHL’ pitaya pulps were collected to investigate the changes in the total phenol, total flavonoid, ascorbate, starch, soluble sugar, major sugar, and organic acid components. Subsequently, the transcription abundance of candidate genes related to ascorbate synthesis and regeneration, starch synthesis and degradation, major sugar synthesis and TCA cycle were analyzed in the three fruit developmental stages of ‘WCHL’ and ‘YCHL’ pitaya pulps.

## 2. Materials and Methods

### 2.1. Plant Materials

**Figure 1:**
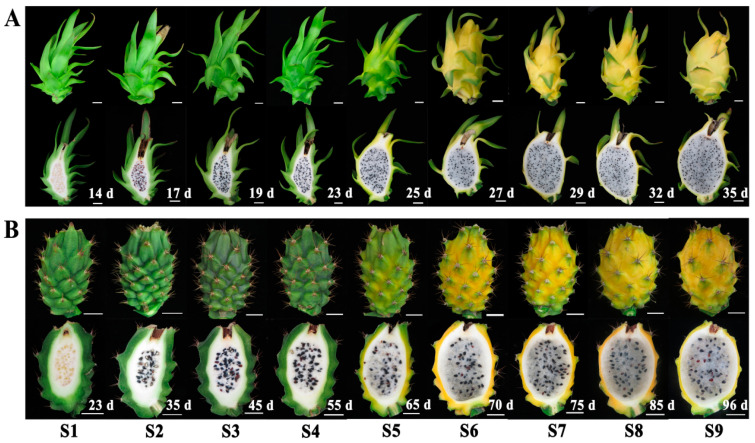
Photograph of ‘WCHL’ (**A**) and ‘YCHL’ (**B**) pitayas at nine (S1–S9) developmental stages.

Two yellow-peel pitayas i.e., Wucihuanglong (*H. undatus*, yellow peel with white flesh (‘WCHL’)) and Youcihuanglong (*H. megalanthus*, yellow peel with white flesh, (‘YCHL’)) were used as materials. The pulps of nine developmental stages (S1-S9) of ‘WCHL’ (14, 17, 19, 23, 25, 27, 29, 32, and 35 day after flowering (DAF)) and ‘YCHL’ (23, 35, 45, 55, 65, 70, 75, 85, and 96 DAF) (Figure 1) from different plants were used for analyses of sugar and organic acid. Pulps from three key fruit development stages of ‘WCHL’ (17, 25 and 29 DAF) and ‘YCHL’ (35, 65 and 75 DAF) pitaya were used for RNA-Seq with three biological repetitions (PRJNA797242). All samples were immediately frozen in liquid nitrogen and stored at −80 °C until use.

### 2.2. Measurements of Total Phenols

Total phenols were measured by the Folin–Ciocalteu method [33]. A total of 0.5 g samples were extracted with 10 mL 80% aqueous methanol (*v*/*v*) solution. After centrifugation at 5000 rpm for 10 min, 20 µL supernatants with 1.8 mL foline-phenol (0.2 mol·L^−1^) were incubated at room temperature for 5 min, and then incubated with 1.2 mL 15% Na_2_(CO)_3_ in 40 °C for 30 min and measured at 760 nm through a spectrophotometer (Infinite M200, Tecan Co., Ltd., Shanghai, China).

### 2.3. Measurements of Total Flavonoids

The total flavonoids were measured by aluminum chloride [33]. A total of 0.5 g samples were extracted with 10 mL 80% aqueous methanol (*v*/*v*) solution. After centrifugation at 5000 rpm for 10 min, 100 µL supernatants, 1.9 mL 90% aqueous ethanol (*v*/*v*) and 300 µL 5% NaNO_2_ were stilled for 6 min. After that, 300 µL 10% Al(NO_3_)_3_ and 2 mL 4% NaOH were added to each mixture with an interval of 6 min, and measured by spectrophotometer at 510 nm after standing 10 min. All determinations were performed in three biological repetitions.

### 2.4. Measurements of Starch and Soluble Sugar

Samples of 0.5 g were extracted with 8 mL 80% aqueous methanol (*v*/*v*) solution and put in a water bath at 80 °C for 30 min. The supernatants were collected after centrifugation at 5000 rpm for 10 min. A total of 10 mg activated carbon (AC) was added to the supernatants and then incubated in a water bath at 80 °C for 30 min. After filtrating the AC, the supernatants were diluted to 25 mL with distilled water. The supernatants and residues were collected for measuring soluble sugar and starch, respectively.

A total of 2 mL distilled water was added to the residues (dried in 80 °C) and kept in boiling water for 10 min. After cooling, 2 mL 9.2 mol·L^−1^ perchloric acid and 6 mL distilled water were added with an interval of 10 min. The supernatants were collected by centrifuging at 5000 rpm for 10 min. The resides were subjected to a similar second extraction, and then the supernatants were collected for analyses of the absorbance values at 620 nm by a spectrophotometer.

A total of 1 mL supernatants with 5 mL anthrone (1 µg·mL^−1^) were kept in boiling water for 10 min, and then the absorbance values were measured at 620 nm by a spectrophotometer.

### 2.5. Measurements of Sugars and Organic Acids

A gas chromatography-mass spectrometry (GC-MS) was used to assay sugars and organic acids according to Lisec et al. [34] with minor modifications. Samples of 50 mg were extracted with 1.4 mL 70% aqueous methanol (*v*/*v*) (20 °C). Then, 60 µL ribitol (0.2 mg·mL^−1^, internal quantitative standard) was added to each mixture and followed by shaking in a Thermomixer Compact (Eppendorf, Germany) at 70 °C with the speed of 950 rpm for 10 min. The supernatants were collected by centrifugation at 11,000× *g* for 10 min. A total of 750 µL chloroform and 1.5 mL distilled water were added to the supernatants and then centrifuged at 2200× *g* for 15 min. A total of 100 µL of the upper phase (polar phase) was dried in a 1.5 mL tube by a vacuum concentrator (Eppendorf Concentrator plus, Germany) at room temperature. The dried samples were oximated with 20 µL of methoxyamination reagent in 37 °C for 2 h in a Thermomixer Compact (950 rpm) and the derivatization reaction without samples was used as the control. After oximation, silylation occurred by adding 35 µL of MSTFA to each mixture and incubated in a Thermomixer Compact (950 rpm) at 37 °C for 30 min. A total of 40 µL of each derivatization product were transferred into glass vials for GC-MS analyses using an Agilent 7890A GC system equipped with an Agilent 7693 autosampler and Agilent 5975C-inert MSD with Triple Axis Detector (Agilent, Atlanta, GA, USA). The operating parameters were performed according to Hua et al. [3].

### 2.6. RNA Extraction and Sequencing

Three key fruit development stages (S2, S5, and S7) of ‘WCHL’ and ‘YCHL’ pitaya pulps were used for RNA-Seq with three biological repetitions (PRJNA797242). The total RNA was extracted using the RNA Prep Pure Plant Kit (TIANGEN, Beijing, China) according to the manufacturer’s instructions. The integrity was assessed using the RNA Nano 6000 Assay Kit of the Bioanalyzer 2100 system (Agilent Technologies, Santa Clara, CA, USA). A total of 1 µg total RNA of each sample was used as input material for the RNA sample preparations. The first-strand cDNA was synthesized using random hexamer primer and M-MuLV Reverse Transcriptase (RNase H-). The second-strand cDNA was synthesized using DNA Polymerase I and RNase H. The library fragments were purified with AMPure XP system (Beckman Coulter, Beverly, MA, USA) to select cDNA fragments of preferentially 370~420 bp in length. PCR was performed with Phusion High-Fidelity DNA polymerase, Universal PCR primers and Index (X) Primer. The library quality was assessed using the Agilent Bioanalyzer 2100 system (Agilent, Santa Clara, CA, USA). The clustering of the index-coded samples was performed on a cBot Cluster Generation System using TruSeq PE Cluster Kit v3-cBot-HS (Illumia, San Diego, CA, USA) according to the manufacturer’s instructions. After cluster generation, the library preparations were sequenced on an Illumina Novaseq platform and 150 bp paired-end reads were generated.

### 2.7. Transcriptome Data Analyses

Raw data (raw reads) of FASTQ format were firstly processed through in-house perl scripts. Clean data (clean reads) were obtained by removing reads containing adapter, reads containing ploy-N and low-quality reads from raw data. Q20, Q30, and GC content of the clean data were calculated and listed in Table S1. The datasets were functionally annotated by pitaya genome using Hisat2 (version 2.0.5) [35]. The quantification of each gene expression level (fragments per kilobase millions (FPKM)) was counted using featureCounts (version 1.5.0-p3). The differential expression analyses of two libraries were performed using the DESeq2 package (version 3.10) of R software (version 4.1.2). Kyoto Encyclopedia of Genes and Genomes (KEGG) enrichment analyses were performed using KofamKOALA (https://www.genome.jp/tools/kofamkoala/, accessed on 5 February 2022). The transcript abundance of candidate genes was drawn by Tbtools software [36]. The accuracy of the RNA-Seq data was verified by RT-qPCR with specific primers (Table S2) according to the method of Xie et al. [32].

### 2.8. Statistical Analysis

Comparisons of the groups were performed by a one-way analysis of variance (One-way ANOVA) with a Duncan test (*p* < 0.01). The correlation coefficients were calculated by the SPSS 25 software (SPSS Inc., Chicago, IL, USA) through Pearson’s correlation and a two-tailed *t* test (*p* < 0.05 and *p* < 0.01).

## 3. Results

### 3.1. Changes in Total Phenol, Total Flavonoid and Ascorbic Acid Contents during Fruit Development

**Figure 2:**
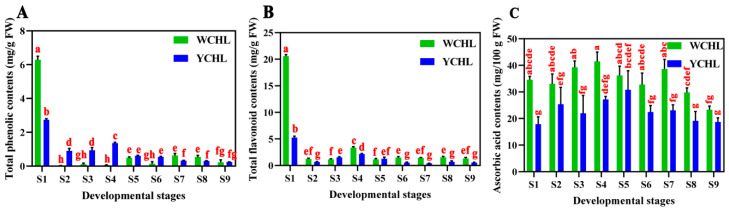
Changes in contents of total phenol (**A**), flavonoid (**B**), and ascorbic acid (**C**) in pulps during fruit developmental stages of ‘WCHL’ and ‘YCHL’ pitayas. Data represent the mean ± S.E. of three biological replicates. Lowercase is indicated the comparison between groups using one-way ANOVA (Duncan test, *p* < 0.01).

Phenol, flavonoid, and ascorbic acid belong to the bioactive compounds which play important roles in plant antioxidant activity [37]. The change in total phenol, total flavonoid, and ascorbic acid contents were analyzed during fruit development of ‘WCHL’ and ‘YCHL’ pitayas. Total phenol and flavonoid contents reached their maximum at stage 1 during fruit development of ‘WCHL’ and ‘YCHL’ pitayas (Figure 2A,B). Higher contents of total phenols and flavonoids were detected in ‘WCHL’ pitaya than that of ‘YCHL’ pitaya at ripening stages. Meanwhile, higher contents of ascorbic acid were detected in ‘WCHL’ pitaya than that of ‘YCHL pitaya (Figure 2C). These data suggested that ‘WCHL’ pitaya accumulates more bioactive compounds than that of ‘YCHL’ pitaya.

### 3.2. Changes in Sugar Contents during Fruit Development

**Figure 3:**
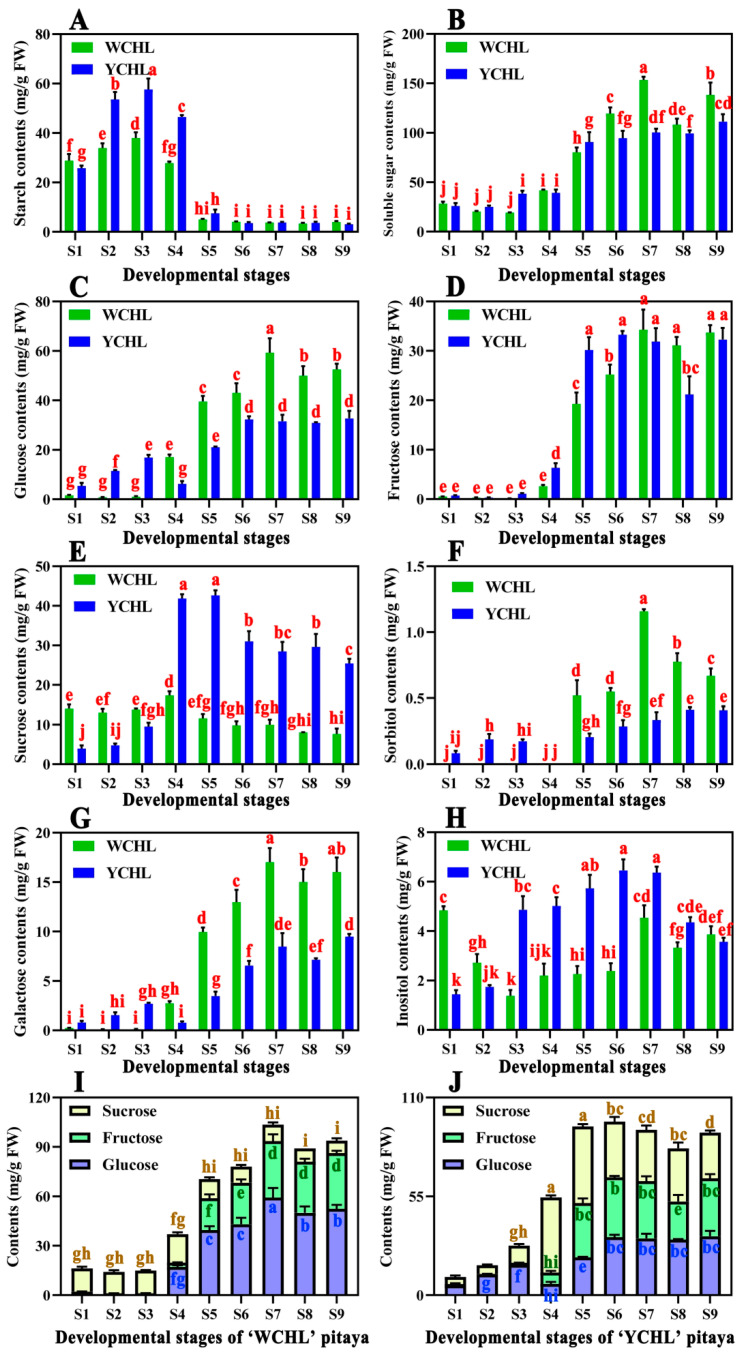
Changes in sugar contents in pulps during fruit developmental stages of ‘WCHL’ and ‘YCHL’ pitayas. (**A**) Starch contents; (**B**) soluble sugar contents; (**C**) glucose contents; (**D**) fructose contents; (**E**) sucrose contents; (**F**) sorbitol contents; (**G**) galactose contents; (**H**) inositol contents; (**I**) major sugar contents in pulps during fruit developmental stages of ‘WCHL’ pitaya; (**J**) major sugar contents in pulps during fruit developmental stages of ‘YCHL’ pitaya. Data represent the mean ± S.E. of three biological replicates. Lowercase indicates the comparison between groups using one-way ANOVA (Duncan test, *p* < 0.01).

Sugar contents were analyzed in pulps during fruit development of ‘WCHL’ and ‘YCHL’ pitayas. Starch showed an increasing pattern from stage 1 to stage 3, and declined from stage 3 to stage 5 and kept low levels thereafter (Figure 3A), suggesting that starch was synthesized from stage 1 to stage 3 and degraded after stage 3. Soluble sugar showed an upward trend and higher contents were detected in pulps of ‘WCHL’ pitaya than that of ‘YCHL’ pitayas during fruit development (Figure 3B). These results suggested that starch was mainly accumulated before stage 4 while soluble sugar was highly accumulated after stage 5, indicating that starch synthesis and degradation resulted in the accumulation of soluble sugars during fruit development of the two pitaya cultivars.

The sugar components including glucose, sucrose, fructose, galactose, inositol, and sorbitol were assayed during fruit development of the two yellow-peel pitayas (Figure 3C–H). Glucose, fructose, sorbitol, and galactose kept increasing tendency while sucrose and inositol began to highly accumulated after stage 5 during fruit maturation. Sucrose, fructose, and glucose were the dominant soluble sugars that highly accumulated at the later stages (from stage 5 to stage 9) in ‘YCHL’ pitayas (Figure 3J), while the main soluble sugar was glucose in ‘WCHL’ pitayas (Figure 3I). These results suggested that the sweetness of ‘YCHL’ pitaya is mainly determined by sucrose, fructose, and glucose compared with only glucose for ‘WCHL’ pitaya.

### 3.3. Changes in Organic Acids during Pitaya Fruit Development

**Figure 4:**
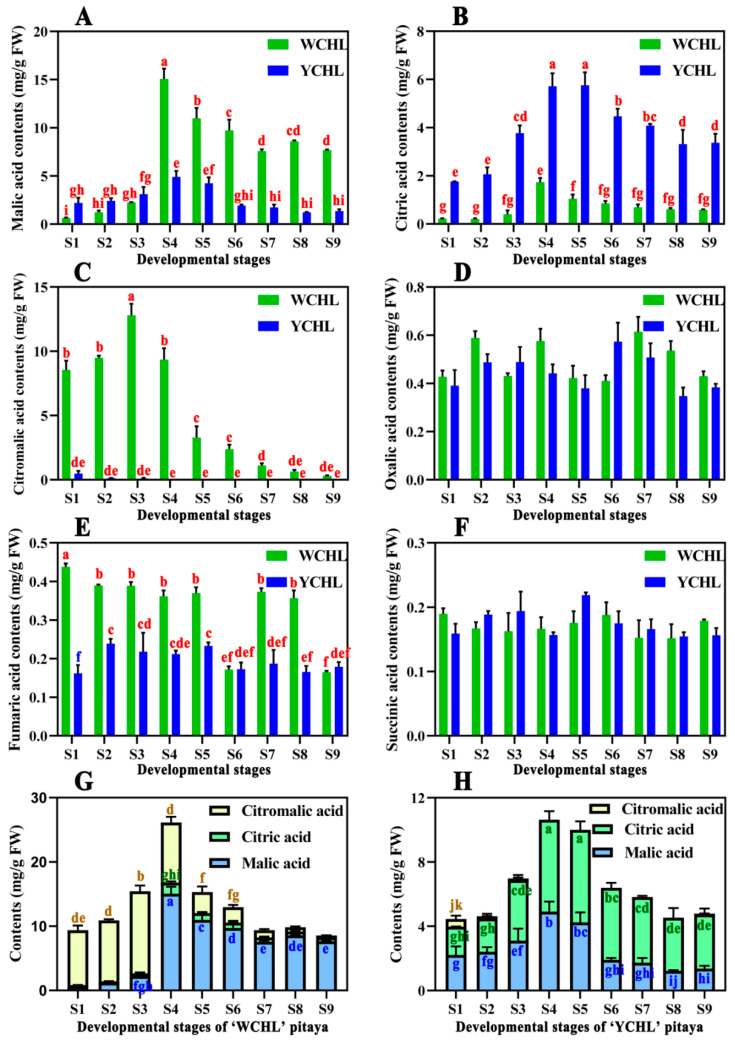
Changes in organic acid contents in pulps during fruit developmental stages of ‘WCHL’ and ‘YCHL’ pitayas. (**A**) Malic acid contents; (**B**) citric acid contents; (**C**) citromalic acid contents; (**D**) oxalic acid contents; (**E**) fumaric acid contents; (**F**) succinic acid contents; (**G**) main acid contents in pulps during fruit developmental stages of ‘WCHL’ pitaya; (**H**) main acid contents in pulps during fruit developmental stages of ‘YCHL’ pitaya. Data represent the mean ± S.E. of three biological replicates. Lowercase indicates the comparison between groups using one-way ANOVA (Duncan test, *p* < 0.01).

The changes in six organic acids, i.e., oxalic, malic, fumaric, succinic, citric, and citramalic acids during fruit development of ‘WCHL’ and ‘YCHL’ pitayas were presented in Figure 4. Malic and citric acid contents were increasing until stage 4 and then gradually decreased during pitaya fruit development of ‘WCHL’ and ‘YCHL’ pitayas (Figure 4A,B). However, citramalic acids were mainly accumulated in the unripe stages and rapidly degraded in the ripe stages of ‘WCHL’ pitaya (Figure 4C). Higher contents of malic, citramalic and fumaric acid were detected in pulps of ‘WCHL’ pitaya than that of ‘YCHL’ pitaya while citric acid was higher accumulated in ‘YCHL’ pitaya than that of ‘WCHL’ pitaya (Figure 4A,C,E). Oxalic and succinic acids kept at lowly levels without significant difference between two pitayas (Figure 4D,F). Thus malic and citric acids were respectively the dominant organic acids in ‘WCHL’ and ‘YCHL’ pitayas, and contribute to sourness of pitaya pulps (Figure 4G,H).

### 3.4. Analyses of Differentially Expressed Genes (DEGs)

**Figure 5:**
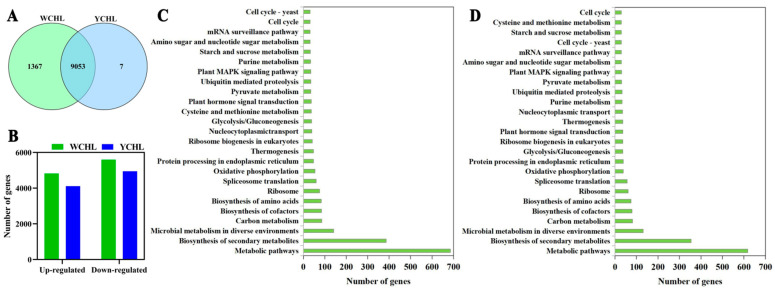
DEGs analyses of ‘WCHL’ and ‘YCHL’ pitaya pulps during three developmental stages. (**A**), Comparison of DEGs between ‘WCHL’ and ‘YCHL’ pitaya pulps; (**B**), the number of up- and down-regulated genes in ‘WCHL’ and ‘YCHL’ pitaya pulps; (**C**,**D**), the KEGG enrichment analysis of DEGs from ‘WCHL’ (**C**) and ‘YCHL’ (**D**) pitaya pulps.

A total of 6.45 G, 6.42 G, 6.50 G, 6.37 G, 6.78 G, and 6.53 G clean data were obtained from 17 d (S2), 25 d (S5), and 29 d (S7) of ‘WCHL’ and 35 d (S2), 65 d (S5), and 75 d (S7) of ‘YCHL’ pitaya pulps, respectively (Table S1). Based on the analyses of differentially expressed genes (DEGs) (padj ≤ 0.05), totally 10,420 and 9060 DEGs were respectively found in the three developmental stages of ‘WCHL’ and ‘YCHL’ pitaya pulps. Among them, most genes (9053 DEGs) were differentially expressed both in ‘WCHL’ and ‘YCHL’ pitaya pulps during fruit maturation (Figure 5A). A total of 4823 and 4111 DEGs were up-regulated while 5597 and 4949 DEGs were down-regulated in ‘WCHL’ and ‘YCHL’ pitayas, respectively (Figure 5B). Moreover, most genes were enriched in the metabolic pathway, especially for secondary metabolites of sugar and acid, including carbon metabolism, glycolysis/gluconeogenesis, and starch and sugar metabolism (Figure 5C,D). Besides, the Pearson’s correlation coefficient R2 between RNA-seq results and RT-qPCR results was 0.8078, which was higher than the threshold of 0.7, indicating that the FPKM values from RNA-Seq is reliable and can be used for subsequent experiments (Figure S1).

### 3.5. Candidate Genes Involved in Ascorbic Acid Metabolism

**Figure 6:**
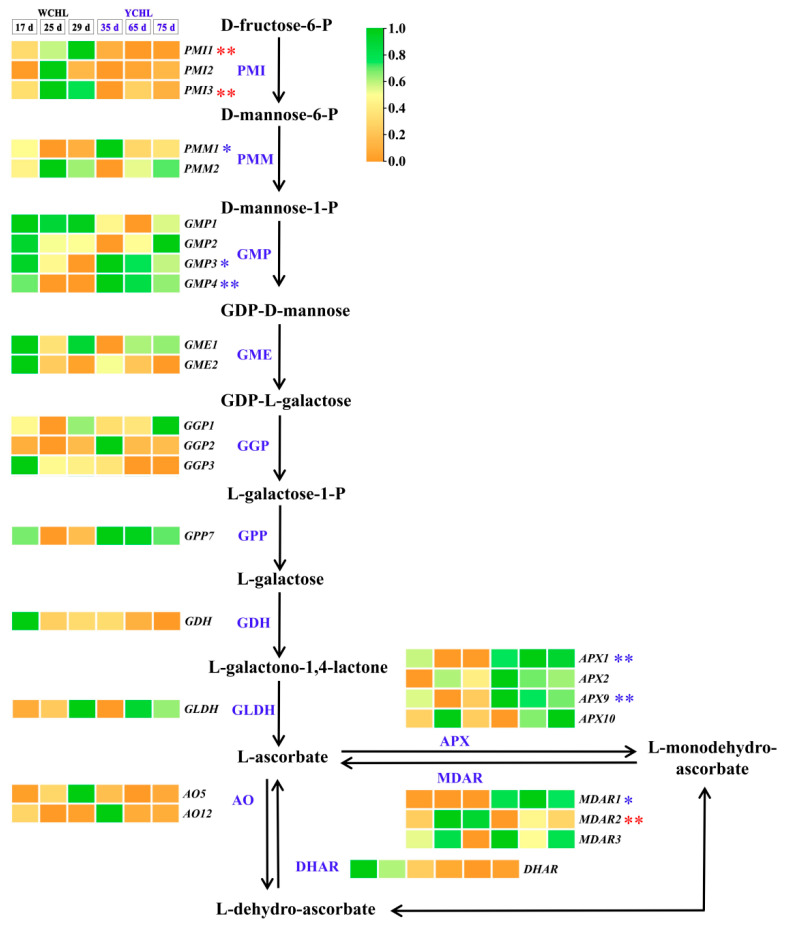
The transcript abundance of candidate genes involved in the proposed L-galactose pathway of ascorbate biosynthesis and regeneration during fruit maturation of ‘WCHL’ and ‘YCHL’ pitayas. Gene abbreviations are shown in Table S3. The correlation analyses of ascorbic acid contents and gene expressions are labeled as * (*p* < 0.05, two-tailed) or ** (*p* < 0.01, two-tailed), red indicates positive relationship while blue is negative.

Based on the ascorbic acid biosynthesis and regeneration pathway, candidate genes encoding its key enzymes were investigated in ‘WCHL’ and ‘YCHL’ pitayas. The L-galactose, L-gulose, myo-inositol and D-galacturonate pathways were proposed to be involved in ascorbate biosynthesis in plants [5,38]. In the two yellow-peel pitayas, all enzymes associated with the L-galactose pathway were found and 61 candidate genes were obtained from the pathway (Table S3). Among these candidate genes, 27 genes were highly expressed in fruit development of yellow-peel pitayas, especially *APX2* and *MDAR3* that showed a downward trend in ‘YCHL’ pitayas and highly expressed at stage 5 in ‘WCHL’ pitaya (Figure 6; Table S3). According to the correlation analyses between the ascorbic acid contents and expression patterns of these candidate gene, *PMM1*, *GMP3*, *GMP4*, *APX1*, *APX9,* and *MDAR1* were down-regulated during fruit development, and showed negative correlation relationship with ascorbic acid contents (Figure 6; Table S4). *PMI1*, *PMI3* and *MDAR2* were up-regulated during fruit development and showed significant positive correlation relationship with ascorbic acid contents. These results indicated that *PMI1* and *PMI3* are the potential key genes involved in ascorbic acid synthesis, while *APXs* (*APX1*, *APX2,* and *APX9*), and *MDARs* (*MDAR1*, *MDAR2,* and *MDAR3*) are responsible for ascorbic acid regeneration in ‘WCHL’ and ‘YCHL’ pitayas.

### 3.6. Candidate Genes Involved in Starch Metabolism

**Figure 7:**
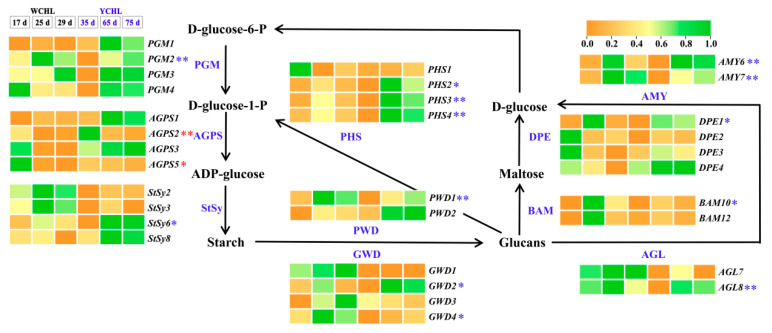
The transcript abundance of candidate genes involved in the proposed starch synthesis and degradation pathways during fruit maturation of ‘WCHL’ and ‘YCHL’ pitayas. Gene abbreviations are shown in Table S5. The correlation analyses of starch content and gene expressions are labeled as * (*p* < 0.05, two-tailed) or ** (*p* < 0.01, two-tailed), red indicates positive relationship while blue is negative.

The starch synthesis and degradation pathway has been proposed in plants [5]. A total of 90 candidate genes involved in starch synthesis and degradation were obtained in pitayas (Table S5). Among them, 32 candidate genes were highly expressed in pulps, especially five starch degradation related genes (*AMY6*, *BAM10*, *BAM12*, *PHS2,* and *PHS3*) which highly expressed at stage 5 during fruit development (Figure 7). A total of 13 candidate genes showed significantly negative correlation, including *PGM2*, *StSy6*, *GWD2*, *GWD4*, *PWD1*, *BAM10*, *DPE1*, *AGL8*, *AMY6*, *AMY7*, *PHS2*, *PHS3,* and *PHS4* (Table S6). Additionally, the expression of *AGPS2* and *AGPS5* exhibited a downward trend and showed positive correlation with starch contents during fruit development. These data demonstrated that *AGPSs* (*AGPS2* and *AGPS5*) are key genes related to the starch synthesis while *AMY6*, *BAM10*, *PHS2,* and *PHS3* are important genes involved in starch degradation in pitaya (Table S6).

### 3.7. Candidate Genes Involved in Sugar Metabolism

**Figure 8:**
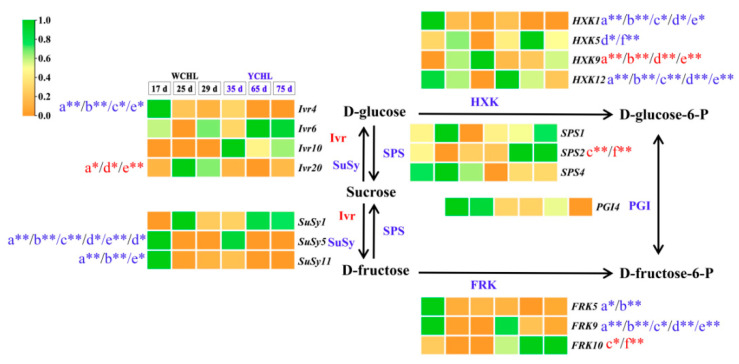
The proposed major sugar synthesis pathways and transcript abundance of their genes in ‘WCHL’ and ‘YCHL’ pitayas. Gene abbreviations are shown in Table S7. Letters a–f represent glucose, fructose, sucrose, sorbitol, galactose and inositol, respectively. The correlation analyses of main sugar contents and gene expressions are labeled as * (*p* < 0.05, two-tailed) or ** (*p* < 0.01, two-tailed), red indicates positive relationship while blue is negative.

Totally, 73 candidate genes associated with sugar metabolism pathway were obtained in the transcriptome data, of which 18 candidate genes (three *SPSs*, four *Ivrs*, three *SuSys*, four *HXKs*, three *FRKs,* and one *PGI*) were highly expressed and probably coordinately regulate the sugar metabolism in the two yellow-peel pitayas (Table S7; Figure 8). Among them, higher expression levels of *SuSy1*, *SuSy5*, *SuSy11*, *FRK5*, *FRK9*, and *FRK10* were detected, of which *SuSy5*, *SuSy11, FRK5*, and *FRK9* showed a downward trend while *SuSy1* and *FRK10* were up-regulated during fruit development. The correlation analyses suggested that up-regulated genes, i.e., *SPS1* and *FRK9* showed positive correlation with sucrose and inositol contents while *Ivr4* and *HXK1* demonstrated positive correlation with glucose, fructose, sorbitol, and galactose accumulation (Figure 8; Table S8). *SuSy5*, *SuSy11*, *Ivr10*, *FRK5*, *FRK10, HXK5*, *HXK9,* and *HXK12* had negative correlation with pitaya main sugars. These results suggested that *SuSys* (*SuSy1*, *SuSy5* and *SuSy11*) and *FRKs* (*FRK5*, *FRK9* and *FRK10*) are key genes to catalyze the pitaya sugar metabolism.

### 3.8. Candidate Genes Associated with Organic Acids Metabolism

**Figure 9:**
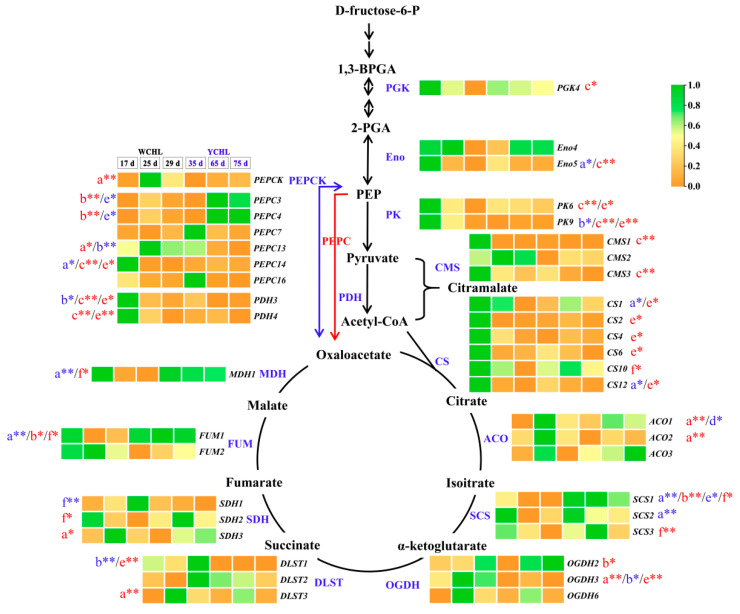
The proposed TCA cycle pathways in yellow pitaya and transcript abundance of their genes in ‘WCHL’ and ‘YCHL’ pitayas. Gene abbreviations are shown in Table S9. Letters a-f represent malic, citric, citromalic, oxalic acid, fumaric, and succinic acids, respectively. The correlation analyses of organic acid contents and gene expressions are labeled as * (*p* < 0.05, two-tailed) or ** (*p* < 0.01, two-tailed), red indicates positive relationship while blue is negative.

The tricarboxylic acid (TCA) cycle was proposed to understand the major organic acids metabolism in yellow-peel pitayas (Figure 9). Totally 92 candidate genes were obtained and 41 genes were highly expressed in pulps (Table S9). The expressions of *PEPC13*, *PEPCK*, *ACO1*, *ACO2*, *DLST3*, *OGDH3,* and *SDH3* were increased and followed with decreasing during fruit development, consisting with the changes in malic acid contents (Table S10). *CS2*, *CS12*, *Eno5*, *FUM1*, *MDH1*, *PEPC14*, *SCS1,* and *SCS2* maintained a decreasing expression pattern and showed negative correlation relationship with malic acid contents (Table S10). *FUM1*, *OGDH2*, *PEPC3,* and *PEPC4* and *SCS1* shared positive correlation relationship with citric acid contents, of which *PEPC3* and *PEPC4* were highly expressed at stage 5 during fruit development with an extremely significant relationship (Table S10). Negative correlations were found between citric acid contents and the expression of *PEPC13*, *PK9*, *PDH3*, *CMS1*, *CMS2*, *CMS3*, *DLST1,* and *OGDH3* with a higher expression level in ‘WCHL’ than ‘YCHL’ (Table S10). These positive and negative correlations with gene expressions were probably the main reason of more citric acid contents in ‘YCHL’ than ‘WCHL’. In addition, only positive correlation relationships were detected between the citromalic acid contents and expression of down-regulated genes, including *PGK4*, *Eno5*, *PK6*, *PK9*, *PDH3*, *PDH4*, *CMS1*, *CMS3*, *PEPC14,* and *CS1*, *CS2*, *CS4*, *CS6*, *CS10,* and *CS12* (Table S10). These results revealed that *PGK4*, *Eno5*, *PK6*, *PK9*, *PDH3*, *PDH4*, *PEPC3*, *PEPC4*, *PEPC13*, *PEPC14,* and *PEPCK* are key upstream genes which limited the carbon sources to enter the TCA cycle. *CMS1*, *CMS2,* and *CMS3* are key genes involved in controlling the citromalic acid biosynthesis and competed acetyl-CoA with TCA cycle. Seventeen genes (*CS1*, *CS2*, *CS4*, *CS6*, *CS10*, *CS12*, *ACO1*, *ACO2*, *SCS1*, *SCS2*, *OGDH2*, *OGDH3*, *DLST1*, *DLST3*, *SDH3*, *FUM1* and *MDH1*) are probably involved in organic acids metabolism of ‘WCHL’ and ‘YCHL’ pitayas.

## 4. Discussions

The metabolites and transcriptome profiling of red-peel and yellow-peel pitayas are used to elucidate a betalain biosynthesis pathway [3,32,39]. Metabolites, including phenol, flavonoid, starch, sugar and acid, are investigated in fruit development of red-peel pitayas [3,4]. The transcriptome analyses of red-peel pitayas showed that the key enzymes (invertase and sucrose synthase) and gene (*HpVAI1*) are involved in the sugar metabolism [16]. Those results provide fundamental information for developing pitaya quality and molecular breeding. However, little information is available about sugar and organic acid metabolism in yellow-peel pitayas. In this study, sugars and organic acids, and expression of metabolism-associated genes during fruit maturation of yellow-peel pitayas were analyzed. DEGs related to sugar and acid metabolism from RNA-Seq were verified by RT-qPCR (Pearson’s correlation coefficient R^2^ = 0.8078; Figure S1).

**Figure 10:**
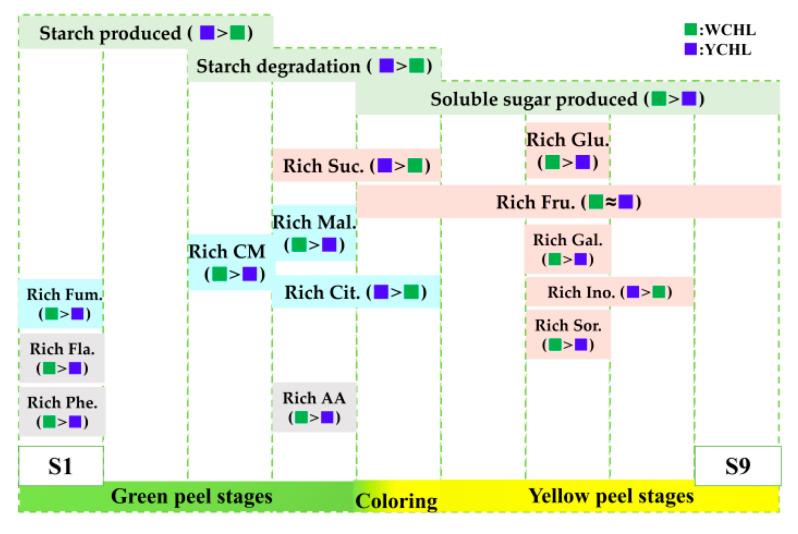
Schematic representation of sugar and organic acid metabolites during fruit developmental stages in two yellow-peel pitayas. Phe: phenolic; Fla: flavonoid; AA: ascorbic acid; Glu: glucose; Suc: sucrose; Fru: fructose; Gal: galactose; Ino: inositol; Sor: sorbitol; Mal: malic; Cit: citric; CM: citromalic; Fum: fumaric.

Phenolics and flavonoids are the antioxidant compounds which can decrease the incidence of oxidative stress and associated with diseases in human health [33]. Phenolics and flavonoids are highly produced in unripe fruits [22] and mature fruits [40]. Besides, ascorbic acid is one of the most abundant antioxidants with various accumulation levels in different species [27,41]. In this study, higher contents of phenols and flavonoids were detected in pulps at the initial fruit development stages compared with low contents in mature stages (Figure 2A,B and Figure 10). A higher content of ascorbic acid was detected in ‘WCHL’ pitaya than that of ‘YCHL’ pitaya (Figure 2C). The L-galactose pathway represents the major route to L-ascorbic acid biosynthesis in higher plants. APX and MDHR are the key components involved in the ascorbate-glutathione cycle which operates in plant chloroplasts for H_2_O_2_ detoxifications [29,42]. In our study, 27 candidate genes involved in ascorbic acids synthesis and regeneration were obtained (Figure 6). *APX2* and *MDAR3* showed higher expression levels than the other genes during fruit development of yellow-peel pitayas (Table S4). Compared with the expression profiling and ascorbic acid contents in yellow-peel pitayas, *MDAR2* were positively regulated the ascorbic acid contents while *APX1*, *APX9,* and *MDAR1* showed negative correlation. These results indicate that APX and MDHR may play major roles in controlling the level of L-ascorbic acid in yellow-peel pitaya fruits and their gene family members were also identified and characterized in *Arabidopsis thaliana* [42,43].

Carbohydrate was first synthesized as starch and then transferred into soluble sugars during fruit development of ‘WCHL’ and ‘YCHL’ pitayas (Figure 3A,B and Figure 10). The starch-to-sugar conversion is not only responsible for fruit sweetness but it also provides energy to coloration [3,44]. APS is a key enzyme of starch synthesis in seeds, tubers, and fruits [45,46,47]. In this work, 32 candidate genes (four *PGMs*, four *AGPSs*, four StSy, four *GWDs*, two *PWDs*, two *AGLs*, *AMYs*, two *BAMs*, four *DPEs,* and four *PHSs*) involved in starch synthesis and degradation were obtained (Figure 7). Expression patterns of most genes involved in starch degradation had a negative correlation with the changes in starch contents (Table S6). *AGPS2* and *AGPS5* kept a downward trend and shared a positive correlation with starch contents during fruit development of yellow-peel pitayas suggesting that AGPS is also a key enzyme for pitaya starch synthesis. AMY and BAM play a prominent role in starch breakdown and participate in the regulation of plant growth, development, and stress responses [48]. PHS participates in phosphorolytic degradation of starch [49]. In the present study, higher expression levels of *AMY6*, *BAM10, PHS2,* and *PHS3* related to starch degradation pathways were detected than other genes and negative correlation with starch contents (Table S5), indicating that these genes probably play important roles in transferring starch into sugar in pitayas.

Sugars and organic acids have strong influence on fruit pH, flavor, and taste [13]. Fructose, glucose, and sucrose were the main abundant sugar components reported in pitaya fruits [3,4,16]. In detail, the major sugar of ‘WCHL’ pitaya was glucose (59.29 mg/g FW) (Figure 3), consistent with the results in red-peel pitayas [3]. However, the dominant sugars of ‘YCHL’ pitaya were sucrose (42.64 mg/g FW), fructose (33.28 mg/g FW), and glucose (32.72 mg/g FW). Most sugars are highly produced at S7 except sucrose which is rich in S4 and S5 of pitayas (Figure 10). The key enzymes, including SPS, SuSy, Ivr, HXK, and FXK, showed various expression patterns for regulating sugar contents during fruit developmental stages [13,16]. In our study, 18 candidate genes (three *SPSs*, three *SuSys*, four *Ivrs*, four *HXKs*, three *FXKs,* and one *PGI*) involved in sugar metabolism were obtained from yellow-peel pitayas (Figure 8). *SPS2*, *Ivr20*, *FRK10,* and *HXK9* had a positive correlation while *SuSy5*, *SuSy11*, *Ivr4*, *FRK5*, *FRK9, HXK1*, *HXK5* and *HXK12* were negatively correlated with sugar accumulation during fruit development of yellow-peel pitayas (Figure 8; Table S8), suggesting that these genes cooperatively regulate sugar synthesis of yellow-peel pitayas.

Malic, citric, and citramalate acids were the three organic acids mainly accumulated at S2 to S5 in pitayas (Figure 10). Due to the degradation of citramalate acid in maturation stages, malic acid (15.06 mg/g FW) and citric acid (5.76 mg/g FW) were the most abundant organic acids in ‘WCHL’ and ‘YCHL’ pitayas, respectively. CMS has been reported as a key enzyme responsible for citramalate synthesis in *Escherichia coli* and apple [50,51]. In yellow-peel pitayas, the expression patterns of *CMS1* and *CMS3* had positive correlation with citromalic acid contents, indicating they probably responsible for citromalic acid biosynthesis of pitaya fruits. PEPC catalyzes the irreversible carboxylation of phosphoenolpyruvate (PEP) to form oxaloacetate which is the substrate for forming citrate [14,52]. In this study, the accumulation of citric acids was significant positive correlation with the expression of *PEPC3* and *PEPC4* during pitaya fruit development. Furthermore, CS catalyzes the reaction of oxaloacetate and acetyl-CoA convert into citrate and coenzyme A, which is the first step in the TCA cycle [52]. However, no *CSs* show correlated relationship with citrate in the two yellow-peel pitayas. This suggests that carbon fluxed into TCA cycle mainly via *PEPC3* and *PEPC4* rather than *CSs*, probably because rich acetyl-CoA fluxed into the irreversible step catalyzed by *CMSs* for citramalate acid biosynthesis. MDH catalyzes the conversion between oxaloacetate and malate and FUM catalyzes the reversible interconversion between malate and fumarate [53,54]. A higher content of malic acid in ‘WCHL’ pitaya were detected than that of ‘YCHL’ pitaya. Malic acid content had a negative correlation with *FUM1* and *MDH1*. Compared with ‘YCHL’ pitaya, *MDH1* was strongly down-regulated in ‘WCHL’ pitaya responsible for more malic acid in ‘WCHL’ pitaya than ‘YCHL’ pitaya. Further work such as enzyme activity analyses and genetic transformation are necessary to elucidate their roles in modulating the fruit quality of pitayas.

## 5. Conclusions

In this study, higher total phenol and flavonoid contents were detected in the stage 1 of yellow-peel pitaya pulps during fruit development. A higher content of ascorbic acid was detected in ‘WCHL’ pitaya than ‘YCHL’ pitaya. Starch was mainly accumulated at early stages and transferred to soluble sugars at S5. Six sugars components (glucose, sucrose, fructose, galactose, inositol, and sorbitol) and six organic acids (oxalic, malic, fumaric, glyceric, succinic, citric and citromalic acid) were detected in pitayas. Glucose and malic acid were the key factors contributed to the taste and flavor quality of ‘WCHL’ pitaya. Sucrose, fructose, and glucose were the main sugars, and citric acid was the dominant acid responsible for fruit quality of ‘YCHL’ pitaya. Based on the expression profilings, 27 candidate genes probably involved in ascorbate biosynthesis and regeneration were achieved, of which *PMIs* was vital for ascorbic acid synthesis, and *APXs* and *MDHRs* played important roles in ascorbate accumulation. A total of 32 candidate genes probably involved in starch synthesis and degradation pathway were isolated, of which *AGPSs* were key genes for starch synthesis and *AMY*, *BAM* and *PHSs* were key regulators controlling starch degradation. *SPSs Ivrs*, *SuSys*, *HXKs*, *FRKs* and *PGI* coordinately regulated sucrose, fructose, and glucose synthesis, of which *SuSys* and *FRKs* were expressed more than the other genes. Organic acids were lowly accumulated in ripening stages associated with downward expression patterns of *PGK*, *Enos*, *PEPCs*, *PKs,* and *PDHs* which limited the carbon flux into TCA cycle. Twenty candidate genes involved in organic acid production and degradation (TCA cycle) were obtained in pitayas. The findings of this study provide basic information for improving fruit quality of yellow-peel pitaya.

## Data Availability

Data is contained within the article.

## Supplementary Materials

The following supporting information can be downloaded at https://www.mdpi.com/article/10.3390/plants11050694/s1, Figure S1: The RT-qPCR analyses of eleven candidate genes (A–K) in sugar and acid metabolic pathway and the coefficient analyses between FPKM values and RT-qPCR of those genes (L); Table S1: RNA-Seq data and corresponding quality control information; Table S2: Specific primers used in RT-qPCR assay; Table S3: The transcript abundance of genes related to ascorbate pathways in ‘WCHL’ and ‘YCHL’ pitayas pulps; Table S4: Correlation analyses between the ascorbic acid contents and expression patterns of candidate genes in ‘WCHL’ and ‘YCHL’ pitayas; Table S5: The transcript abundance of genes related to starch synthesis and degradation pathways in ‘WCHL’ and ‘YCHL’ pitayas; Table S6: Correlation analyses between the starch contents or total soluble sugar and expression patterns of candidate genes in ‘WCHL’ and ‘YCHL’ pitayas; Table S7: The transcript abundance of genes related to major sugar biosynthesis pathways in ‘WCHL’ and ‘YCHL’ pitayas; Table S8: Correlation analyses between the main sugar contents and expression patterns of candidate gene in ‘WCHL’ and ‘YCHL’ pitayas; Table S9: The transcript abundance of genes related to TCA cycle pathways in ‘WCHL’ and ‘YCHL’ pitayas; Table S10: Correlation analyses between the main organic acid contents and expression patterns of candidate genes in ‘WCHL’ and ‘YCHL’ pitayas.
