# Supplementary material for: Metabolic Profiling of Sugars and Organic Acids, and Expression Analyses of Metabolism-Associated Genes in Two Yellow-Peel Pitaya Species
Source: Plants (Basel). 2022 Mar 4;11(5):694. doi: 10.3390/plants11050694 (PMC8912497; doi:10.3390/plants11050694)
Supplement: Supplementary file 1 [file plants-11-00694-s001.zip › plants-1580344-supplementary.pdf]

# Supplementary figures and tables

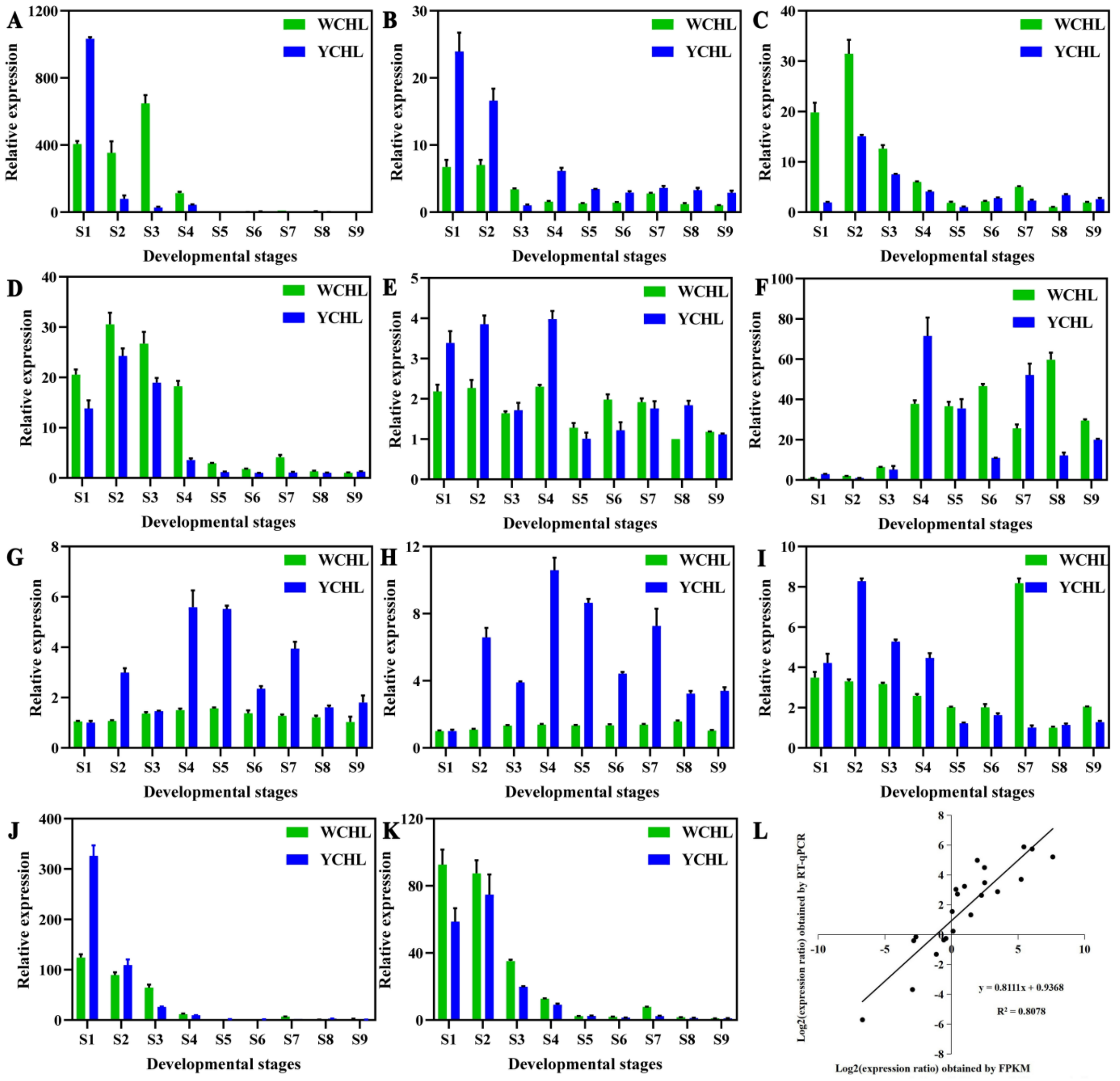


Figure S1. The RT-qPCR analyses of eleven candidate genes (A-K) in sugar and acid metabolic pathway and the coefficient analyses between FPKM values and RT-qPCR of those genes (L). A, *CMS1*; B, *CS6*; C, *FRK5*; D, *FRK9*; E, *MDAR1*; F, *PEPC4*; G, *PHS2*; H, *PHS3*; I, *PMI1*; J, *SuSy5*; K, *SuSy11*.

Table S1 RNA-Seq data and corresponding quality control information

| Samples | Raw reads | Clean reads | Clean_bases (G) | Error rate (%) | Q20 (%) | Q30 (%) | GC content (%) |
|---|---|---|---|---|---|---|---|
| WCHL_17d_1 | 45918120 | 44088506 | 6.61 | 0.02 | 98.14 | 94.31 | 49.64 |
| WCHL_17d_2 | 48264390 | 46108368 | 6.92 | 0.03 | 97.95 | 93.94 | 50.04 |
| WCHL_17d_3 | 40405646 | 38763342 | 5.81 | 0.03 | 97.95 | 93.93 | 49.75 |
| WCHL_25d_1 | 42257762 | 40626122 | 6.09 | 0.02 | 98.22 | 94.51 | 50.66 |
| WCHL_25d_2 | 48071026 | 46752758 | 7.01 | 0.02 | 98.85 | 96.09 | 50.57 |
| WCHL_25d_3 | 43826692 | 40976414 | 6.15 | 0.02 | 98.22 | 94.67 | 49.99 |
| WCHL_29d_1 | 45988084 | 42674020 | 6.4 | 0.02 | 98.14 | 94.46 | 50.01 |
| WCHL_29d_2 | 47180636 | 45564148 | 6.83 | 0.02 | 98.86 | 96.14 | 50.4 |
| WCHL_29d_3 | 46150874 | 41831586 | 6.27 | 0.02 | 98.19 | 94.58 | 50.26 |
| YCHL_35d_1 | 41089912 | 39661570 | 5.95 | 0.02 | 98.12 | 94.28 | 49.27 |
| YCHL_35d_2 | 45494498 | 44206436 | 6.63 | 0.03 | 97.98 | 93.88 | 49.94 |
| YCHL_35d_3 | 45244634 | 43577446 | 6.54 | 0.02 | 98.08 | 94.15 | 51.38 |
| YCHL_65d_1 | 45518136 | 41775096 | 6.27 | 0.02 | 98.33 | 94.9 | 51.09 |
| YCHL_65d_2 | 47612182 | 45114430 | 6.77 | 0.02 | 98.32 | 94.9 | 50.43 |
| YCHL_65d_3 | 50410912 | 48703300 | 7.31 | 0.02 | 98.62 | 95.45 | 49.99 |
| YCHL_75d_1 | 45291970 | 44124280 | 6.62 | 0.02 | 98.84 | 96.02 | 50.7 |
| YCHL_75d_2 | 45216534 | 43936170 | 6.59 | 0.02 | 98.81 | 96 | 50.28 |
| YCHL_75d_3 | 44770172 | 42611336 | 6.39 | 0.03 | 98.06 | 94.06 | 50.47 |

Table S2 Specific primers used in RT-qPCR assay.

| Gene name | Forward primer (5' to 3') | Reverse primer (5' to 3') |
|---|---|---|
| *CMS1* | GTATGAACTTGATGGGCTT | TGGTATTGTGCTTGGAAAA |
| *CS6* | CAGTGGAACATGTGCAAGGC | CTGCTTCCCTGAGTGCTTGA |
| *FRK5* | TGCTGGCGATGCATTTGTTG | CCGGGAATTGCACCTCTCTT |
| *FRK9* | GAGGACGAGCCGAGATTGAG | GCTGGGATTGCTCCCTTCTT |
| *MDAR1* | TGGTCTTGAGCTCAGTGCAG | ATCAGCGGTGAAGAGTCGTG |
| *PEPC4* | CGTGCTCGAGTAGACGAACT | AGCACGTTCACGGGTATTGT |
| *PHS2* | TGAATCCCCCGAGTCCAGAT | GGAAAATCCCTCTGGGGCAA |
| *PHS3* | GCGGAGTGTTTGGCACATTT | ACCACGGCCAAATCCTTCAT |
| *PMI1* | GCTGGCCTAACACCTAAGCA | TGTGCATCCATCACTGCCAT |
| *SuSy5* | GTGAACCTTGTCGTGGTTGC | ATCTGGGCAGAGATCCACCT |
| *SuSy11* | GGAGCTGGACTTTGAGCCAT | TGTTGAGGAACTCGACACCG |

Table S3 The transcript abundance of genes related to ascorbate pathways in 'WCHL' and 'YCHL' pitayas pulps.

| Gene names | Gene ID | 'WCHL' pitaya | | | 'YCHL' pitaya | | |
|---|---|---|---|---|---|---|---|
| | | 17 d | 25 d | 29 d | 35 d | 65 d | 75 d |
| L-ascorbate oxidase | | | | | | | |
| *AO1* | *HU01G01245.1* | 0 | 0 | 0 | 0 | 0 | 0 |
| *AO2* | *HU01G01270.1* | 0 | 0 | 0 | 5.44 | 0 | 0 |
| *AO3* | *HU01G01464.1* | 8.69 | 0 | 0 | 6.17 | 0.77 | 2.43 |
| *AO4* | *HU01G01815.1* | 0 | 0 | 0 | 0 | 0 | 0 |
| *AO5* | *HU02G01327.1* | 3.25 | 11.34 | 32.22 | 7.84 | 2.36 | 4.77 |
| *AO6* | *HU02G02455.1* | 0 | 0 | 0 | 0 | 0 | 0 |
| *AO7* | *HU02G03061.1* | 0 | 0 | 0 | 0 | 0 | 0 |
| *AO8* | *HU03G01567.1* | 0 | 0 | 0 | 0 | 0 | 0 |
| *AO9* | *HU03G01573.1* | 0 | 0 | 0 | 0 | 0 | 0 |
| *AO10* | *HU05G01813.1* | 0 | 0 | 0 | 0 | 0 | 0 |
| *AO11* | *HU05G01868.1* | 0.2 | 0 | 0 | 0 | 0 | 0 |
| *AO12* | *HU06G00762.1* | 21.26 | 1.59 | 4.01 | 70.48 | 7.37 | 8.26 |
| *AO13* | *HU06G00824.1* | 0 | 0 | 0 | 0 | 0 | 0 |
| *AO14* | *HU06G00825.1* | 3.45 | 1.48 | 4.27 | 1.67 | 1.6 | 6.9 |
| *AO15* | *HU07G01170.1* | 1.92 | 0.42 | 0.61 | 1.62 | 1.54 | 0.35 |
| *AO16* | *HU08G00550.1* | 3.53 | 5.73 | 4.62 | 3.04 | 7.24 | 7.32 |
| *AO17* | *HU08G01290.1* | 0.08 | 0 | 0 | 0 | 0 | 0 |
| *AO18* | *HU08G01291.1* | 0.95 | 0 | 0 | 0 | 0.07 | 0 |
| *AO19* | *HU08G01953.1* | 0.96 | 0.17 | 0.04 | 0.46 | 0.2 | 0.18 |
| *AO20* | *HU09G01170.1* | 0 | 0 | 0 | 0 | 0 | 0 |
| *AO21* | *HU09G01171.1* | 0 | 0 | 0 | 0 | 0 | 0 |
| *AO22* | *HU10G01971.1* | 0 | 0 | 0 | 0 | 0 | 0 |
| L-ascorbate peroxidase | | | | | | | |
| *APX1* | *HU01G00039.1* | 74.57 | 46.68 | 47.18 | 82.93 | 94.49 | 90.73 |

| | | | | | | | |
|---|---|---|---|---|---|---|---|
| *APX2* | *HU01G01291.1* | 272.97 | 461.8 | 401.56 | 582.28 | 485.69 | 468.56 |
| *APX3* | *HU03G00392.1* | 12.78 | 15.52 | 17.58 | 8.22 | 5.85 | 4.39 |
| *APX4* | *HU06G00143.1* | 0.13 | 0 | 0 | 0 | 0 | 0 |
| *APX5* | *HU06G00147.1* | 0 | 0 | 0 | 0 | 0 | 0 |
| *APX6* | *HU06G00148.1* | 0 | 0 | 0 | 0 | 0 | 0 |
| *APX7* | *HU06G01014.1* | 0.13 | 0.56 | 0.25 | 1.42 | 0.92 | 0 |
| *APX8* | *HU07G00395.1* | 1.61 | 1.53 | 1.41 | 1.28 | 2.6 | 2.14 |
| *APX9* | *HU07G00844.1* | 44.61 | 12.07 | 26.05 | 71.03 | 56.49 | 52.29 |
| *APX10* | *HU09G00808.1* | 31.33 | 89.6 | 30.18 | 9.96 | 63.5 | 90.36 |
| *APX11* | *HU11G00172.1* | 7.61 | 13.25 | 20.95 | 4.32 | 8.33 | 10.14 |
| *APX12* | *HU11G01184.1* | 10.57 | 16.62 | 22.54 | 14.95 | 23.02 | 26.83 |
| dehydroascorbate reductase | | | | | | | |
| *DHAR* | *HU06G01275.1* | 102.27 | 65.84 | 32.58 | 16.54 | 9.7 | 13 |
| L-galactose dehydrogenase | | | | | | | |
| *GDH* | *HU03G00469.1* | 24.04 | 12.52 | 13.43 | 13.47 | 10.16 | 8.41 |
| GDP-L-galactose phosphorylase | | | | | | | |
| *GGP1* | *HU01G01690.1* | 41.04 | 20.95 | 48.5 | 35.26 | 36.66 | 63.54 |
| *GGP2* | *HU05G01762.1* | 28.5 | 21.94 | 32.63 | 87.65 | 32.66 | 33.4 |
| *GGP3* | *HU08G00238.1* | 69.97 | 42.44 | 40 | 36.62 | 17.32 | 17.65 |
| L-galactono-1,4-lactone dehydrogenase | | | | | | | |
| *GLDH* | *HU10G01073.1* | 11.24 | 12.57 | 19.61 | 10.46 | 18.74 | 16.38 |
| GDP-mannose 3,5-epimerase | | | | | | | |
| *GME1* | *HU03G01059.1* | 25.03 | 15.77 | 23.54 | 10.66 | 19.54 | 20 |
| *GME2* | *HU08G01094.1* | 71.84 | 44.86 | 37.8 | 54.27 | 43.83 | 36.27 |
| Mannose-1-phosphate guanylyltransferase | | | | | | | |
| *GMP1* | *HU05G01570.1* | 11.46 | 10.73 | 11.32 | 7.84 | 4.77 | 8.47 |
| *GMP2* | *HU05G01571.1* | 8.69 | 5.98 | 5.95 | 2.59 | 5.88 | 9.27 |
| *GMP3* | *HU05G01623.1* | 10.73 | 7.09 | 3.47 | 11.19 | 9.49 | 8.02 |
| *GMP4* | *HU06G00539.1* | 56.89 | 26.42 | 26.47 | 70.57 | 63.34 | 55.22 |
| L-galactose-1-phosphate phosphatase | | | | | | | |

| | | | | | | | |
|---|---|---|---|---|---|---|---|
| *GPP1* | *HU01G00275.1* | 13.31 | 19.77 | 11.13 | 8.38 | 5.54 | 7.14 |
| *GPP2* | *HU01G02287.1* | 13.25 | 8.34 | 8.19 | 8.7 | 5.94 | 8.13 |
| *GPP3* | *HU02G02148.1* | 19.08 | 9.6 | 8.81 | 13.62 | 11.31 | 13.3 |
| *GPP4* | *HU10G01800.1* | 0.08 | 0 | 0.28 | 0 | 0 | 0 |
| *GPP5* | *HU10G01801.1* | 0.11 | 0 | 0.17 | 0.4 | 0.16 | 0 |
| *GPP6* | *HU10G01803.1* | 2.41 | 1.91 | 2.1 | 2.97 | 1.56 | 2.94 |
| *GPP7* | *HU10G02033.1* | 103.78 | 42.02 | 57.55 | 132.68 | 129.92 | 106.29 |
| Monodehydroascorbate reductase | | | | | | | |
| *MDAR1* | *HU02G02424.1* | 10.25 | 9.6 | 9.42 | 39.76 | 47.38 | 38.15 |
| *MDAR2* | *HU05G00113.1* | 38.84 | 79.42 | 74.26 | 25.12 | 49.93 | 40.96 |
| *MDAR3* | *HU09G00954.1* | 200.19 | 239.18 | 126.62 | 263.93 | 194.2 | 237.34 |
| Mannose-6-phosphate isomerase | | | | | | | |
| *PMI1* | *HU05G01684.1* | 14.47 | 21.35 | 31.8 | 9.15 | 6.5 | 7.23 |
| *PMI2* | *HU05G01685.1* | 0.06 | 8.58 | 1.19 | 0 | 0.5 | 1.18 |
| *PMI3* | *HU06G00668.1* | 13.9 | 33.02 | 27.09 | 4.03 | 11.9 | 7.27 |
| Phosphomannomutase | | | | | | | |
| *PMM1* | *HU07G00464.1* | 13.77 | 3.11 | 5.42 | 25.14 | 9.71 | 11 |
| *PMM2* | *HU09G00757.1* | 29.33 | 47.97 | 35.85 | 14.85 | 32.63 | 38.5 |

Table S4 Correlation analyses between the ascorbic acid contents and expression patterns of candidate genes in ‘WCHL’ and ‘YCHL’ pitayas.

| Gene names | Gene ID | ascorbic acid |
|---|---|---|
| *AO5* | *HU02G01327.1* | 0.4 |
| *AO12* | *HU06G00762.1* | -0.311 |
| *APX1* | *HU01G00039.1* | -0.751** |
| *APX2* | *HU01G01291.1* | -0.294 |
| *APX9* | *HU07G00844.1* | -0.676** |
| *APX10* | *HU09G00808.1* | -0.021 |
| *DHAR* | *HU06G01275.1* | 0.391 |
| *GDH* | *HU03G00469.1* | 0.266 |
| *GGP1* | *HU01G01690.1* | -0.006 |
| *GGP2* | *HU05G01762.1* | -0.036 |
| *GGP3* | *HU08G00238.1* | 0.404 |
| *GLDH* | *HU10G01073.1* | 0.153 |
| *GME1* | *HU03G01059.1* | 0.429 |
| *GME2* | *HU08G01094.1* | -0.084 |
| *GMP1* | *HU05G01570.1* | 0.132 |
| *GMP2* | *HU05G01571.1* | 0.128 |
| *GMP3* | *HU05G01623.1* | -0.497* |
| *GMP4* | *HU06G00539.1* | -0.603** |
| *GPP7* | *HU10G02033.1* | -0.366 |
| *MDAR1* | *HU02G02424.1* | -0.531* |
| *MDAR2* | *HU05G00113.1* | 0.669** |
| *MDAR3* | *HU09G00954.1* | -0.368 |
| *PMI1* | *HU05G01684.1* | 0.687** |
| *PMI2* | *HU05G01685.1* | 0.358 |
| *PMI3* | *HU06G00668.1* | 0.761** |
| *PMM1* | *HU07G00464.1* | -0.577* |
| *PMM2* | *HU09G00757.1* | 0.388 |

Note: The data was analyzed using SPSS 25 with Pearson's correlation. The significant positive correlation coefficients are indicated in red values while negative correlation coefficients are indicated in blue values.

* indicates correlation significant at the 0.05 level ($p < 0.05$, two-tailed) while ** indicates correlation significant at the 0.01 level ($p < 0.01$, two-tailed).

Table S5 The transcript abundance of genes related to starch synthesis and degradation pathways in 'WCHL' and 'YCHL' pitayas.

| Gene names | Gene ID | 'WCHL' pitaya | | | 'YCHL' pitaya | | |
|---|---|---|---|---|---|---|---|
| | | 17 d | 25 d | 29 d | 35 d | 65 d | 75 d |
| Alpha-glucosidase | | | | | | | |
| *AGL1* | *HU02G01725.1* | 1.02 | 0.67 | 0.33 | 2.96 | 3.66 | 4.94 |
| *AGL2* | *HU02G01726.1* | 1.13 | 1.04 | 0.73 | 5.49 | 6.44 | 6.1 |
| *AGL3* | *HU02G01727.1* | 8.19 | 5 | 6.53 | 7.22 | 5.14 | 7.53 |
| *AGL4* | *HU02G01728.1* | 1.19 | 0.65 | 0.48 | 0 | 0 | 0 |
| *AGL5* | *HU02G01729.1* | 0.91 | 0.2 | 0.78 | 0.54 | 1.74 | 3.44 |
| *AGL6* | *HU02G01730.1* | 6.93 | 4.74 | 7.04 | 2.39 | 1.26 | 1.81 |
| *AGL7* | *HU03G01386.1* | 23.77 | 26.53 | 26.47 | 16.25 | 21.31 | 16.17 |
| *AGL8* | *HU05G00176.1* | 53.86 | 68.41 | 44.28 | 18.17 | 55.74 | 53.64 |
| *AGL9* | *HU09G01939.1* | 4.59 | 12.68 | 5.42 | 0.81 | 1.53 | 1.24 |
| Glucose-1-phosphate adenylyltransferase | | | | | | | |
| *AGPS1* | *HU01G00286.1* | 14.46 | 24.98 | 22.71 | 25.52 | 79.08 | 66.24 |
| *AGPS2* | *HU03G00147.1* | 52.25 | 10.01 | 14.58 | 123.15 | 28.2 | 18.17 |
| *AGPS3* | *HU03G02063.1* | 63.03 | 14.95 | 12.89 | 50.09 | 68.08 | 76.34 |
| *AGPS4* | *HU06G01322.1* | 0.45 | 0.06 | 0 | 0 | 0.19 | 0.21 |
| *AGPS5* | *HU07G00703.1* | 26.56 | 1.87 | 0.77 | 7.01 | 5.8 | 3.57 |
| Alpha-amylase | | | | | | | |
| *AMY1* | *HU01G00458.1* | 0.55 | 0.8 | 3.03 | 2.52 | 3.16 | 1.68 |
| *AMY2* | *HU01G01364.1* | 4.48 | 6.89 | 13.06 | 1.95 | 3.58 | 4.03 |
| *AMY3* | *HU01G02422.1* | 0.76 | 0.7 | 1.68 | 0.35 | 0.92 | 0.53 |
| *AMY4* | *HU01G02675.1* | 1.19 | 2 | 3.19 | 1.23 | 2.69 | 3.39 |
| *AMY5* | *HU02G00188.1* | 1.99 | 2.4 | 4.39 | 1.33 | 2.44 | 4.13 |
| *AMY6* | *HU02G00293.1* | 38.78 | 210.71 | 76.22 | 20.37 | 220.68 | 194.78 |
| *AMY7* | *HU02G01323.1* | 23.59 | 97.01 | 74.82 | 10.59 | 53.44 | 64.52 |
| *AMY8* | *HU02G02922.1* | 1.6 | 1.74 | 2.69 | 1.27 | 1.04 | 2.24 |

| | | | | | | | |
|---|---|---|---|---|---|---|---|
| *AMY9* | *HU02G03420.1* | 0.62 | 1.16 | 1.63 | 1.11 | 2.11 | 2.88 |
| *AMY10* | *HU03G01593.1* | 0.72 | 0.88 | 2.42 | 0.27 | 0.98 | 1.3 |
| *AMY11* | *HU04G00245.1* | 0.35 | 0.86 | 1.83 | 1.27 | 1.38 | 1.09 |
| *AMY12* | *HU05G00001.1* | 0 | 0.06 | 0 | 0 | 0 | 0 |
| *AMY13* | *HU05G00002.1* | 0 | 0 | 0 | 0 | 0 | 0 |
| *AMY14* | *HU05G01323.1* | 3.9 | 4.3 | 5.25 | 8.14 | 1.16 | 1.45 |
| *AMY15* | *HU05G01802.1* | 0.34 | 0.99 | 2.79 | 0.29 | 1.18 | 1.21 |
| *AMY16* | *HU06G00016.1* | 0.21 | 0 | 0 | 0 | 0 | 0 |
| *AMY17* | *HU06G00973.1* | 0 | 0 | 0 | 0 | 0 | 0 |
| *AMY18* | *HU06G01277.1* | 1.76 | 3.12 | 3.43 | 0.9 | 2.48 | 2.54 |
| *AMY19* | *HU07G00472.1* | 1.25 | 1.65 | 3.12 | 1.98 | 1.45 | 0.68 |
| *AMY20* | *HU07G00886.1* | 0.68 | 1.05 | 4.06 | 0.54 | 1.53 | 1.63 |
| *AMY21* | *HU07G00912.1* | 0 | 0.09 | 0.15 | 0.06 | 0.04 | 0.04 |
| *AMY22* | *HU07G00913.1* | 0.14 | 0.56 | 0.42 | 0.2 | 0.57 | 1.16 |
| *AMY23* | *HU07G01462.1* | 0 | 0 | 0 | 0 | 0 | 0 |
| *AMY24* | *HU07G01511.1* | 55.71 | 0.24 | 0.09 | 12.41 | 0 | 0 |
| *AMY25* | *HU08G00353.1* | 0.12 | 0 | 0 | 0 | 0 | 0 |
| *AMY26* | *HU08G00466.1* | 2.22 | 3.97 | 10.3 | 2.68 | 14.69 | 12.55 |
| *AMY27* | *HU08G00990.1* | 0.56 | 0.68 | 2.55 | 2.68 | 3.84 | 4.42 |
| *AMY28* | *HU08G01940.1* | 0.49 | 1.01 | 2.44 | 1.35 | 0.5 | 1.39 |
| *AMY29* | *HU09G01705.1* | 0.51 | 0.42 | 1.27 | 0.2 | 0.32 | 0.22 |
| *AMY30* | *HU10G00319.1* | 0.68 | 0.07 | 0.07 | 0.2 | 0.07 | 0 |
| *AMY31* | *HU10G01279.1* | 0.13 | 0.18 | 0.22 | 1.17 | 2.01 | 2.14 |
| *AMY32* | *HU11G00819.1* | 0.43 | 1.78 | 3.17 | 0.81 | 1.01 | 1.3 |
| *AMY33* | *HU11G01508.1* | 0.17 | 1.22 | 0.23 | 0 | 0 | 0.34 |
| *AMY34* | *HU11G01790.1* | 1.8 | 2.36 | 7.11 | 1.62 | 2.26 | 2.37 |
| Beta-amylase | | | | | | | |
| *BAM1* | *HU03G02052.1* | 2.13 | 0.56 | 0.2 | 3.58 | 0 | 0 |

| | | | | | | | |
|---|---|---|---|---|---|---|---|
| *BAM2* | *HU03G02053.1* | 3.09 | 0.4 | 0 | 9.6 | 0 | 0 |
| *BAM3* | *HU04G00443.1* | 9.08 | 2.36 | 2.17 | 17.96 | 2.96 | 1.88 |
| *BAM4* | *HU04G00659.1* | 11.07 | 15.76 | 25.91 | 5.14 | 12.22 | 10.96 |
| *BAM5* | *HU05G00342.1* | 1.43 | 2.91 | 3.68 | 0.82 | 3.08 | 2.93 |
| *BAM6* | *HU07G00441.1* | 5.67 | 2.67 | 4.73 | 2.36 | 1.52 | 3.56 |
| *BAM7* | *HU08G00306.1* | 7.01 | 11.06 | 6.13 | 6.53 | 9.31 | 7 |
| *BAM8* | *HU09G00508.1* | 0.19 | 0 | 0 | 0 | 0 | 0 |
| *BAM9* | *HU09G01694.1* | 0 | 0 | 0 | 0 | 0 | 0 |
| *BAM10* | *HU10G00777.1* | 118.01 | 716.89 | 355.9 | 119.42 | 277.19 | 187.61 |
| *BAM11* | *HU10G00778.1* | 31.2 | 67.58 | 55.74 | 26.13 | 75.23 | 51.72 |
| *BAM12* | *HU10G00833.1* | 146.49 | 629.91 | 247.03 | 182.11 | 260.99 | 200.17 |
| *BAM13* | *HU11G01792.1* | 9.46 | 10.69 | 7.48 | 1.32 | 10.6 | 10.89 |
| *BAM14* | *HU11G01794.1* | 8.99 | 10.12 | 8.06 | 9.02 | 10.4 | 10.84 |
| 4-alpha-glucanotransferase | | | | | | | |
| *DPE1* | *HU09G01260.1* | 11.66 | 28.02 | 12.14 | 10.49 | 22.64 | 21.32 |
| *DPE2* | *HU09G01976.1* | 4.62 | 1.46 | 1.78 | 0.72 | 1.68 | 1.39 |
| *DPE3* | *HU09G01977.1* | 2.35 | 1.14 | 0.82 | 1.18 | 1.71 | 1.46 |
| *DPE4* | *HU10G01164.1* | 29.09 | 24.17 | 15.91 | 28.99 | 37.07 | 37.2 |
| Glucan, water dikinase | | | | | | | |
| *GWD1* | *HU01G01312.1* | 39.35 | 47.53 | 61.78 | 0 | 0 | 0 |
| *GWD2* | *HU02G00177.1* | 28.76 | 51.71 | 24.07 | 16.49 | 69.88 | 60.45 |
| *GWD3* | *HU0G00205.1* | 2.72 | 8.4 | 12.69 | 7.38 | 6.07 | 4.92 |
| *GWD4* | *HU10G00183.1* | 17 | 47.68 | 32.45 | 1.53 | 7.98 | 14.5 |
| Phosphoglucomutase | | | | | | | |
| *PGM1* | *HU02G01892.1* | 15.49 | 18.08 | 15.74 | 19.89 | 37.96 | 30.94 |
| *PGM2* | *HU09G00757.1* | 29.33 | 47.97 | 35.85 | 14.85 | 32.63 | 38.5 |
| *PGM3* | *HU10G00720.1* | 13.27 | 13.25 | 15.56 | 10.88 | 15.77 | 15.3 |
| *PGM4* | *HU10G00736.1* | 30.72 | 18.75 | 18.22 | 10.86 | 27.99 | 25.43 |
| Glucan phosphorylase | | | | | | | |

| | | | | | | | |
|---|---|---|---|---|---|---|---|
| *PHS1* | *HU02G00384.1* | 21.33 | 3.81 | 7.97 | 5.41 | 6.11 | 8.15 |
| *PHS2* | *HU06G01230.1* | 60.24 | 140.56 | 80.34 | 29.64 | 338.64 | 210.85 |
| *PHS3* | *HU06G01231.1* | 73.35 | 209.78 | 109.27 | 42.98 | 374.6 | 273.23 |
| *PHS4* | *HU07G02193.1* | 42.73 | 82.33 | 50.14 | 19.11 | 150.08 | 115.6 |
| Phosphoglucan, water dikinase | | | | | | | |
| *PWD1* | *HU09G02021.1* | 16.79 | 44.53 | 34.75 | 10.33 | 24.01 | 32.25 |
| *PWD2* | *HU09G02105.1* | 1.81 | 5.13 | 4.37 | 3.35 | 9.24 | 9.78 |
| Starch synthase | | | | | | | |
| *StSy1* | *HU02G03120.1* | 18.3 | 15.84 | 15.87 | 16.13 | 14.04 | 15.85 |
| *StSy2* | *HU05G01186.1* | 24.34 | 35.77 | 28.49 | 8.42 | 14.06 | 13.16 |
| *StSy3* | *HU05G01187.1* | 38.95 | 57.58 | 46.68 | 20.36 | 31.67 | 20.99 |
| *StSy4* | *HU05G02284.1* | 13.98 | 12.78 | 11.62 | 6.24 | 10.92 | 12.19 |
| *StSy5* | *HU06G02646.1* | 10.56 | 18.33 | 10.32 | 6.94 | 12.34 | 12.98 |
| *StSy6* | *HU07G01224.1* | 17.25 | 20.69 | 18.86 | 15.4 | 24.95 | 24.71 |
| *StSy7* | *HU09G00010.1* | 3.16 | 0.73 | 0 | 1.73 | 0 | 0 |
| *StSy8* | *HU10G00589.1* | 45.6 | 51.86 | 15.35 | 46.61 | 101.75 | 90.26 |
| *StSy9* | *HU10G01954.1* | 2.15 | 1.83 | 1.33 | 2 | 2.88 | 3.83 |
| *StSy10* | *HU10G01957.1* | 4.69 | 4.86 | 4.47 | 2.67 | 4.9 | 5.32 |

Table S6 Correlation analyses between the starch contents or total soluble sugar and expression patterns of candidate genes in ‘WCHL’ and ‘YCHL’ pitayas.

| Gene names | Gene ID | Starch |
|---|---|---|
| *AGL7* | *HU03G01386.1* | -0.406 |
| *AGL8* | *HU05G00176.1* | -0.655** |
| *AGPS1* | *HU01G00286.1* | -0.379 |
| *AGPS2* | *HU03G00147.1* | 0.890** |
| *AGPS3* | *HU03G02063.1* | 0.213 |
| *AGPS5* | *HU07G00703.1* | 0.522* |
| *AMY6* | *HU02G00293.1* | -0.689** |
| *AMY7* | *HU02G01323.1* | -0.832** |
| *BAM10* | *HU10G00777.1* | -0.520* |
| *BAM12* | *HU10G00833.1* | -0.426 |
| *DPE1* | *HU09G01260.1* | -0.578* |
| *DPE2* | *HU09G01976.1* | 0.101 |
| *DPE3* | *HU09G01977.1* | 0.18 |
| *DPE4* | *HU10G01164.1* | 0.072 |
| *GWD1* | *HU01G01312.1* | -0.29 |
| *GWD2* | *HU02G00177.1* | -0.589* |
| *GWD3* | *HU0G00205.1* | -0.162 |
| *GWD4* | *HU10G00183.1* | -0.550* |
| *PGM1* | *HU02G01892.1* | -0.291 |
| *PGM2* | *HU09G00757.1* | -0.835** |
| *PGM3* | *HU10G00720.1* | -0.246 |
| *PGM4* | *HU10G00736.1* | -0.221 |
| *PHS1* | *HU02G00384.1* | 0.278 |
| *PHS2* | *HU06G01230.1* | -0.587* |
| *PHS3* | *HU06G01231.1* | -0.636** |
| *PHS4* | *HU07G02193.1* | -0.651** |
| *PWD1* | *HU09G02021.1* | -0.793** |
| *PWD2* | *HU09G02105.1* | -0.439 |
| *StSy2* | *HU05G01186.1* | -0.436 |
| *StSy3* | *HU05G01187.1* | -0.401 |
| *StSy6* | *HU07G01224.1* | -0.517* |
| *StSy8* | *HU10G00589.1* | -0.205 |

Table S7 The transcript abundance of genes related to major sugar biosynthesis pathways in 'WCHL' and 'YCHL' pitayas.

| Gene names | Gene ID | 'WCHL' pitaya | | | 'YCHL' pitaya | | |
|---|---|---|---|---|---|---|---|
| | | 17 d | 25 d | 29 d | 35 d | 65 d | 75 d |
| Fructokinase | | | | | | | |
| *FRK1* | *HU02G00407.1* | 10.97 | 10.51 | 13.56 | 10.63 | 11 | 19.75 |
| *FRK2* | *HU02G00410.1* | 0.49 | 0.18 | 1.19 | 0.27 | 0.32 | 0.83 |
| *FRK3* | *HU02G00412.1* | 0.39 | 0.28 | 0.34 | 1.88 | 0.87 | 1.23 |
| *FRK4* | *HU02G01173.1* | 14.8 | 27.63 | 35.05 | 5.41 | 18.75 | 24.92 |
| *FRK5* | *HU02G01891.1* | 113.54 | 19.18 | 23.79 | 22.62 | 9.19 | 16.56 |
| *FRK6* | *HU02G03173.1* | 5.05 | 3.12 | 2.53 | 0.83 | 0.58 | 1.12 |
| *FRK7* | *HU02G03267.1* | 3.03 | 0.55 | 0 | 20.62 | 4.53 | 10.88 |
| *FRK8* | *HU05G01760.1* | 9.48 | 2.1 | 4.12 | 12.37 | 9.04 | 8.98 |
| *FRK9* | *HU06G02152.1* | 271.62 | 33.4 | 24.63 | 235.29 | 73.1 | 42.2 |
| *FRK10* | *HU06G02213.1* | 171.04 | 124.8 | 122.54 | 240.79 | 321.37 | 323.58 |
| *FRK11* | *HU07G01671.1* | 0 | 0 | 0 | 0 | 0 | 0.09 |
| Hexokinase | | | | | | | |
| *HXK1* | *HU01G00828.1* | 28.98 | 6.16 | 2.64 | 6.68 | 1.35 | 1.38 |
| *HXK2* | *HU02G01230.1* | 6.26 | 1.87 | 5.67 | 14.24 | 14.24 | 18.24 |
| *HXK3* | *HU02G01231.1* | 4.01 | 5.6 | 14.36 | 0.85 | 3.52 | 7 |
| *HXK4* | *HU03G01733.1* | 6.03 | 8.83 | 13.04 | 8.36 | 5.02 | 5.95 |
| *HXK5* | *HU03G02729.1* | 28.13 | 36.44 | 21.24 | 31.02 | 44.5 | 32.77 |
| *HXK6* | *HU05G00008.1* | 12.06 | 10.64 | 14.91 | 3.79 | 4.77 | 4.29 |
| *HXK7* | *HU06G00308.1* | 0.07 | 0 | 0 | 0.57 | 2.1 | 1.26 |
| *HXK8* | *HU08G00363.1* | 2.86 | 6.13 | 17.46 | 2.82 | 9.21 | 17.44 |
| *HXK9* | *HU08G01226.1* | 6.67 | 31.06 | 46.67 | 13.27 | 15.69 | 29.31 |
| *HXK10* | *HU0G00093.1* | 0 | 0.15 | 0.14 | 0.5 | 1.47 | 1.15 |
| *HXK11* | *HU0G00094.1* | 1 | 0 | 1.85 | 0 | 1.16 | 3.34 |
| *HXK12* | *HU11G00500.1* | 42.44 | 35.63 | 19.52 | 45.91 | 34.39 | 25.44 |

Invertase

| | | | | | | | |
|---|---|---|---|---|---|---|---|
| *Ivr1* | *HU01G00073.1* | 0 | 0 | 0 | 0 | 0 | 0 |
| *Ivr2* | *HU01G00074.1* | 0 | 0 | 0 | 0 | 0.12 | 0 |
| *Ivr3* | *HU02G01252.1* | 29.19 | 20.81 | 23.08 | 20.24 | 12.11 | 12.04 |
| *Ivr4* | *HU03G00349.1* | 80.56 | 21.89 | 14.64 | 27.68 | 7.28 | 5.84 |
| *Ivr5* | *HU03G00845.1* | 3.36 | 3.09 | 4.04 | 2.37 | 4.19 | 4.04 |
| *Ivr6* | *HU03G01461.1* | 29.78 | 11.53 | 32.53 | 20.73 | 42.48 | 39.5 |
| *Ivr7* | *HU03G02454.1* | 1.05 | 0 | 0.11 | 0 | 0.14 | 0 |
| *Ivr8* | *HU03G02455.1* | 0.68 | 0.15 | 0 | 2.59 | 0 | 0.11 |
| *Ivr9* | *HU03G02456.1* | 0.05 | 0 | 0 | 0 | 0.25 | 0.72 |
| *Ivr10* | *HU03G02457.1* | 1.71 | 1.78 | 1.69 | 41.92 | 19.49 | 27.16 |
| *Ivr11* | *HU04G00751.1* | 0.07 | 0 | 0 | 0 | 0 | 0 |
| *Ivr12* | *HU04G00755.1* | 0 | 0 | 0 | 0 | 0 | 0 |
| *Ivr13* | *HU05G00322.1* | 0 | 0 | 0 | 0 | 0 | 0 |
| *Ivr14* | *HU07G00113.1* | 0.06 | 0 | 0 | 0 | 0 | 0 |
| *Ivr15* | *HU07G00148.1* | 0.79 | 0 | 0 | 0 | 0 | 0 |
| *Ivr16* | *HU07G00250.1* | 18.28 | 17.17 | 18.8 | 16.75 | 12.69 | 11.33 |
| *Ivr17* | *HU07G00349.1* | 7.97 | 0.95 | 0.77 | 0.42 | 0 | 0 |
| *Ivr18* | *HU07G02013.1* | 0.04 | 0.67 | 0.3 | 0.64 | 0.74 | 0.77 |
| *Ivr19* | *HU07G02022.1* | 0.28 | 0.72 | 1.14 | 1.57 | 2.13 | 1.42 |
| *Ivr20* | *HU08G00679.1* | 18.92 | 32.39 | 27.19 | 18.86 | 16.86 | 19.31 |
| *Ivr21* | *HU08G01394.1* | 2.38 | 0.88 | 2.27 | 0.17 | 0.81 | 0.92 |
| *Ivr22* | *HU08G01398.1* | 0 | 0 | 0 | 0 | 0 | 0 |
| *Ivr23* | *HU11G00399.1* | 2.36 | 0 | 0.41 | 13.61 | 2.96 | 2.02 |
| *Ivr24* | *HU11G01556.1* | 12.94 | 7.29 | 4.22 | 5.79 | 4.44 | 3.78 |
| *Ivr25* | *HU11G01647.1* | 0.14 | 0.9 | 0.07 | 0 | 0.05 | 0 |
| *Ivr26* | *HU11G01656.1* | 0 | 0 | 0 | 0 | 0 | 0 |
| *Ivr27* | *HU11G01657.1* | 0.87 | 0 | 0 | 1.43 | 0 | 0 |

| | | | | | | | |
|---|---|---|---|---|---|---|---|
| Glucose-6-phosphate isomerase | | | | | | | |
| *PGI1* | *HU01G02580.1* | 2.13 | 1.45 | 0.28 | 1.39 | 2.2 | 1.01 |
| *PGI2* | *HU01G02694.1* | 5.57 | 8.04 | 10.2 | 18.69 | 5.49 | 11.6 |
| *PGI3* | *HU04G01930.1* | 0.57 | 1.1 | 2.93 | 1.05 | 0.2 | 0.31 |
| *PGI4* | *HU05G00170.1* | 52.99 | 50.53 | 37.64 | 37.41 | 42.92 | 31.51 |
| *PGI5* | *HU05G00393.1* | 3.29 | 1.42 | 0.6 | 2.09 | 2.13 | 2.71 |
| *PGI6* | *HU07G00352.1* | 10.42 | 7.38 | 7.44 | 5.27 | 8.79 | 9.15 |
| *PGI7* | *HU07G00362.1* | 2.54 | 6.62 | 3.88 | 2.13 | 4.54 | 3 |
| *PGI8* | *HU07G01113.1* | 14.88 | 17.14 | 17.81 | 15.86 | 19.27 | 16.26 |
| Sucrose-phosphate synthase | | | | | | | |
| *SPS1* | *HU03G00894.1* | 20.77 | 27.66 | 15.06 | 20.59 | 21.48 | 24.69 |
| *SPS2* | *HU05G00771.1* | 58.92 | 42.4 | 51.66 | 50.28 | 78.01 | 77.51 |
| *SPS3* | *HU09G02070.1* | 0.06 | 0.02 | 0.02 | 0 | 0 | 0 |
| *SPS4* | *HU11G00237.1* | 20.08 | 25.17 | 16.84 | 2.78 | 10.12 | 9.76 |
| Sucrose synthase | | | | | | | |
| *SuSy1* | *HU02G00890.1* | 50 | 296.75 | 109.37 | 117.09 | 258.88 | 236.68 |
| *SuSy2* | *HU02G01574.1* | 4.58 | 17.4 | 0.35 | 3.48 | 0.14 | 0.48 |
| *SuSy3* | *HU02G02087.1* | 9.65 | 11.78 | 12.71 | 7.95 | 11.97 | 16.78 |
| *SuSy4* | *HU03G00958.1* | 1.71 | 3.24 | 4.83 | 0.88 | 2.57 | 3.25 |
| *SuSy5* | *HU03G02498.1* | 146.58 | 4.67 | 3.9 | 130.53 | 2.26 | 1.96 |
| *SuSy6* | *HU03G02967.1* | 28.37 | 53.1 | 33.85 | 8.47 | 9.62 | 9.13 |
| *SuSy7* | *HU05G00839.1* | 13.21 | 19.9 | 18.85 | 7.55 | 13.29 | 12.64 |
| *SuSy8* | *HU07G01441.1* | 10.82 | 55.53 | 57.16 | 3.61 | 6.03 | 6.63 |
| *SuSy9* | *HU07G01442.1* | 6.11 | 24.03 | 33.17 | 2.77 | 3.4 | 4.45 |
| *SuSy10* | *HU08G01008.1* | 0.42 | 0.42 | 0.51 | 0.29 | 0 | 0 |
| *SuSy11* | *HU11G00421.1* | 353.84 | 33.53 | 62.84 | 83.81 | 26.09 | 21.84 |

Table S8 Correlation analyses between the main sugar contents and expression patterns of candidate gene in 'WCHL' and 'YCHL' pitayas.

| Gene names | Gene ID | Glucose | Fructose | Sucrose | Sorbitol | Galactose | Inositol |
|---|---|---|---|---|---|---|---|
| *FRK5* | *HU02G01891.1* | -0.564* | -0.616** | -0.356 | -0.394 | -0.444 | -0.296 |
| *FRK9* | *HU06G02152.1* | -0.821** | -0.875** | -0.565* | -0.642** | -0.777** | -0.349 |
| *FRK10* | *HU06G02213.1* | -0.321 | 0.199 | 0.546* | -0.457 | -0.368 | 0.692** |
| *HXK1* | *HU01G00828.1* | -0.625** | -0.709** | -0.475* | -0.473* | -0.531* | -0.337 |
| *HXK5* | *HU03G02729.1* | -0.263 | 0.11 | 0.186 | -0.491* | -0.387 | 0.646** |
| *HXK9* | *HU08G01226.1* | 0.796** | 0.593** | 0.254 | 0.766** | 0.817** | -0.106 |
| *HXK12* | *HU11G00500.1* | -0.741** | -0.783** | -0.610** | -0.698** | -0.751** | -0.217 |
| *Ivr4* | *HU03G00349.1* | -0.600** | -0.731** | -0.514* | -0.428 | -0.510* | -0.406 |
| *Ivr6* | *HU03G01461.1* | -0.025 | 0.225 | 0.442 | 0.004 | -0.01 | 0.325 |
| *Ivr10* | *HU03G02457.1* | -0.321 | -0.168 | 0.053 | -0.327 | -0.361 | 0.117 |
| *Ivr20* | *HU08G00679.1* | 0.587* | 0.201 | -0.22 | 0.561* | 0.600** | -0.378 |
| *PGI4* | *HU05G00170.1* | -0.278 | -0.389 | -0.455 | -0.277 | -0.286 | -0.148 |
| *SPS1* | *HU03G00894.1* | -0.126 | -0.04 | -0.06 | -0.311 | -0.151 | 0.156 |
| *SPS2* | *HU05G00771.1* | -0.166 | 0.385 | 0.718** | -0.322 | -0.183 | 0.765** |
| *SPS4* | *HU11G00237.1* | 0.282 | 0.066 | -0.215 | 0.258 | 0.328 | -0.2 |
| *SuSy1* | *HU02G00890.1* | 0.284 | 0.454 | 0.295 | 0.021 | 0.186 | 0.434 |
| *SuSy5* | *HU03G02498.1* | -0.761** | -0.908** | -0.642** | -0.569* | -0.699** | -0.495* |
| *SuSy11* | *HU11G00421.1* | -0.607** | -0.666** | -0.396 | -0.426 | -0.496* | -0.316 |

Table S9 The transcript abundance of genes related to TCA cycle pathways in 'WCHL' and 'YCHL' pitayas.

| Gene names | Gene ID | 'WCHL' pitaya | | | 'YCHL' pitaya | | |
|---|---|---|---|---|---|---|---|
| | | 17 d | 25 d | 29 d | 35 d | 65 d | 75 d |
| Aconitate hydratase | | | | | | | |
| *ACO1* | *HU01G01718.1* | 35.63 | 84.96 | 54.41 | 47.16 | 70.54 | 63.8 |
| *ACO2* | *HU01G01740.1* | 25.97 | 57.35 | 28.71 | 11.55 | 25.87 | 18.51 |
| *ACO3* | *HU08G00194.1* | 21.99 | 30.09 | 20.84 | 26.33 | 27.45 | 32.12 |
| 2-isopropylmalate synthase | | | | | | | |
| *CMS1* | *HU01G02605.1* | 173.29 | 0.89 | 0.89 | 4.33 | 0 | 0 |
| *CMS2* | *HU06G01828.1* | 22.46 | 32.57 | 28.19 | 7.79 | 16.09 | 14.98 |
| *CMS3* | *HU08G00711.1* | 67.82 | 32.98 | 22.81 | 31.18 | 15.46 | 10.87 |
| Citrate synthase | | | | | | | |
| *CS1* | *HU01G00929.1* | 153.83 | 130.68 | 65.25 | 80.49 | 120.89 | 90.4 |
| *CS2* | *HU02G00183.1* | 53.36 | 6.07 | 7.12 | 8.69 | 12.68 | 7.84 |
| *CS3* | *HU02G03260.1* | 22.37 | 24.66 | 21.63 | 17.04 | 14.58 | 13.29 |
| *CS4* | *HU03G01582.1* | 62.81 | 46.63 | 39.22 | 37.82 | 42.27 | 39.99 |
| *CS5* | *HU04G00898.1* | 0.31 | 1.11 | 0.92 | 0 | 9.98 | 10.05 |
| *CS6* | *HU06G02202.1* | 88.34 | 29.9 | 32.27 | 48.52 | 36.3 | 24.6 |
| *CS7* | *HU07G00576.1* | 0 | 0 | 0 | 0 | 0 | 0 |
| *CS8* | *HU07G00635.1* | 0.86 | 0.63 | 0.27 | 0.23 | 0.16 | 0.15 |
| *CS9* | *HU07G01303.1* | 11.49 | 15.74 | 14.41 | 33.19 | 12.33 | 20.86 |
| *CS10* | *HU08G00662.1* | 59.65 | 48.16 | 29.09 | 46.04 | 53.3 | 42.8 |
| *CS11* | *HU08G01468.1* | 7.52 | 0.55 | 0.17 | 11.63 | 2.03 | 1.9 |
| *CS12* | *HU10G00190.1* | 97.88 | 10.18 | 3.46 | 30.99 | 12.43 | 6.87 |
| *CS13* | *HU11G01933.1* | 0.43 | 0 | 0 | 1.08 | 0 | 0 |
| *CS14* | *HU11G01978.1* | 0 | 0 | 0 | 0.34 | 0 | 0 |
| Dihydrolipoyllysine-residue succinyltransferase | | | | | | | |
| *DLST1* | *HU01G01093.1* | 0.34 | 0.2 | 0.59 | 0 | 0 | 0 |
| *DLST2* | *HU08G00217.1* | 0.76 | 0.49 | 1.79 | 1.37 | 1.25 | 0.82 |

| | | | | | | | |
|---|---|---|---|---|---|---|---|
| *DLST3* | *HU10G01974.1* | 74.68 | 106.46 | 84.94 | 78.33 | 95.45 | 78.05 |
| Enolase | | | | | | | |
| *Eno1* | *HU02G01933.1* | 0 | 0.19 | 0.15 | 4.56 | 8.97 | 7.68 |
| *Eno2* | *HU04G01426.1* | 73.53 | 10.45 | 13.18 | 27.58 | 21.15 | 17.49 |
| *Eno3* | *HU07G02203.1* | 22.9 | 16.26 | 17.92 | 27.08 | 21.53 | 22.65 |
| *Eno4* | *HU09G00620.1* | 285.93 | 309.69 | 100.97 | 144.21 | 276.51 | 271.38 |
| *Eno5* | *HU09G00621.1* | 623.52 | 201.32 | 132.05 | 315.07 | 181.19 | 174.38 |
| Fumarate hydratase | | | | | | | |
| *FUM1* | *HU02G00881.1* | 42.42 | 25.84 | 29.08 | 42.32 | 44.18 | 43.09 |
| *FUM2* | *HU06G01498.1* | 31.19 | 33.24 | 27.3 | 20.55 | 23.94 | 26.85 |
| Malate dehydrogenase | | | | | | | |
| *MDH1* | *HU06G02301.1* | 482.2 | 174.33 | 152.57 | 467.01 | 425.44 | 397.99 |
| *MDH2* | *HU07G00587.1* | 13.47 | 1.7 | 0.69 | 28.87 | 2.19 | 3.01 |
| *MDH3* | *HU11G01942.1* | 1.17 | 0 | 0 | 0.85 | 0.29 | 0.54 |
| *MDH4* | *HU11G01943.1* | 0.56 | 0 | 0 | 0.39 | 0.39 | 0.99 |
| 2-oxoglutarate dehydrogenase | | | | | | | |
| *OGDH1* | *HU01G01093.1* | 0.34 | 0.2 | 0.59 | 0 | 0 | 0 |
| *OGDH2* | *HU01G02567.1* | 15.67 | 18.05 | 36.54 | 5.6 | 37.22 | 45.16 |
| *OGDH3* | *HU04G02070.1* | 58.94 | 92.45 | 77.58 | 38.57 | 45.66 | 37.77 |
| *OGDH4* | *HU06G01674.1* | 0.42 | 0.96 | 0.3 | 0.07 | 1.17 | 2.67 |
| *OGDH5* | *HU08G00217.1* | 0.76 | 0.49 | 1.79 | 1.37 | 1.25 | 0.82 |
| *OGDH6* | *HU10G01974.1* | 74.68 | 106.46 | 84.94 | 78.33 | 95.45 | 78.05 |
| Pyruvate dehydrogenase | | | | | | | |
| *PDH1* | *HU02G00259.1* | 0.82 | 0.06 | 0.5 | 2.62 | 4.11 | 3.29 |
| *PDH2* | *HU02G02870.1* | 24.87 | 32.76 | 34.45 | 28.84 | 35.09 | 33.3 |
| *PDH3* | *HU07G00191.1* | 109.24 | 35.16 | 25.44 | 39.63 | 19.4 | 21.42 |
| *PDH4* | *HU10G00282.1* | 99.89 | 58.41 | 43.15 | 48.42 | 52.11 | 43.38 |
| Phosphoenolpyruvate carboxylase | | | | | | | |
| *PEPC1* | *HU01G00450.1* | 0.88 | 0 | 0 | 0 | 0 | 0 |
| *PEPC2* | *HU01G00894.1* | 25.5 | 51.62 | 21.3 | 15.52 | 58.34 | 56.87 |

| | | | | | | | |
|---|---|---|---|---|---|---|---|
| *PEPC3* | *HU01G00987.1* | 5.88 | 170.05 | 43.39 | 4.45 | 620.03 | 513.18 |
| *PEPC4* | *HU01G00988.1* | 2.9 | 86.75 | 22.02 | 3.08 | 314.72 | 315.79 |
| *PEPC5* | *HU01G00989.1* | 0.57 | 2.35 | 3.06 | 0.1 | 0.88 | 0.72 |
| *PEPC6* | *HU01G00990.1* | 2.88 | 2.27 | 1.77 | 39.71 | 72.07 | 48.41 |
| *PEPC7* | *HU01G00991.1* | 13.77 | 7.7 | 21.5 | 104.68 | 24.4 | 14.17 |
| *PEPC8* | *HU02G00490.1* | 1.96 | 1.7 | 1.51 | 0.08 | 0.19 | 0.19 |
| *PEPC9* | *HU02G02553.1* | 1.49 | 0.7 | 0.81 | 0.57 | 0.37 | 0.37 |
| *PEPC10* | *HU02G02555.1* | 0.1 | 0 | 0.05 | 1.08 | 0.78 | 0.26 |
| *PEPC11* | *HU02G02556.1* | 4.74 | 3.1 | 3.57 | 1.17 | 1.58 | 2.08 |
| *PEPC12* | *HU02G03351.1* | 0.06 | 0.63 | 0.91 | 0.21 | 0.79 | 0 |
| *PEPC13* | *HU03G01008.1* | 102.25 | 181.3 | 125.63 | 120.33 | 35.27 | 21.17 |
| *PEPC14* | *HU03G01874.1* | 295.76 | 93.13 | 87.12 | 103.75 | 116.19 | 103.5 |
| *PEPC15* | *HU04G00337.1* | 0 | 0 | 0 | 0 | 0 | 0 |
| *PEPC16* | *HU08G01056.1* | 42.11 | 11.62 | 9.38 | 104.78 | 7.23 | 4.34 |
| *PEPC17* | *HU08G02244.1* | 5.42 | 1.96 | 3.23 | 2.36 | 3.35 | 2.95 |
| *PEPC18* | *HU09G00446.1* | 0 | 0 | 0 | 0 | 0 | 0 |
| *PEPC19* | *HU09G00447.1* | 0 | 0 | 0 | 0 | 0.36 | 0 |
| *PEPC20* | *HU09G01073.1* | 9.35 | 8.03 | 8.83 | 5.07 | 6.49 | 8.11 |
| *PEPC21* | *HU10G01889.1* | 0.05 | 0 | 0 | 0 | 0 | 0 |
| *PEPC22* | *HU10G01890.1* | 0.74 | 0.25 | 0.05 | 0.03 | 0 | 0 |
| *PEPC23* | *HU10G02036.1* | 0.04 | 0 | 0 | 0 | 0 | 0 |
| *PEPC24* | *HU10G02037.1* | 0.19 | 0.08 | 0 | 0 | 0 | 0 |
| Phosphoenolpyruvate carboxykinase | | | | | | | |
| *PEPCK* | *HU10G00784.1* | 3.51 | 92.48 | 36.01 | 0.31 | 9.23 | 14.93 |
| Phosphoglycerate kinase | | | | | | | |
| *PGK1* | *HU03G02104.1* | 69.42 | 40.4 | 34.77 | 17.24 | 9.99 | 12.27 |
| *PGK2* | *HU03G02313.1* | 6.06 | 4.5 | 7.25 | 4.8 | 5.79 | 7.91 |
| *PGK3* | *HU05G00547.1* | 23.81 | 20.02 | 24.91 | 10.42 | 35.89 | 34.58 |

| | | | | | | | |
|---|---|---|---|---|---|---|---|
| *PGK4* | *HU06G01772.1* | 546.86 | 359.49 | 135.71 | 397.25 | 366.86 | 338.26 |
| *PGK5* | *HU06G02389.1* | 13.51 | 15.17 | 9.66 | 8.49 | 14.74 | 12.14 |
| *PGK6* | *HU10G01770.1* | 7.56 | 8.18 | 10.63 | 2.86 | 1.81 | 1.64 |
| Pyruvate kinase | | | | | | | |
| *PK1* | *HU01G02685.1* | 0.68 | 0.67 | 2.01 | 1.1 | 0.38 | 0.44 |
| *PK2* | *HU01G02786.1* | 0.31 | 0.21 | 0.66 | 0 | 0.07 | 0.13 |
| *PK3* | *HU02G01707.1* | 37.2 | 22.1 | 24.03 | 13.33 | 11.08 | 12.62 |
| *PK4* | *HU02G02411.1* | 65.91 | 51.51 | 28.16 | 57.65 | 61.36 | 57.94 |
| *PK5* | *HU03G00827.1* | 53.4 | 7.82 | 3.55 | 40.42 | 46.3 | 38.4 |
| *PK6* | *HU03G02352.1* | 164.91 | 107.49 | 70.4 | 105.15 | 101.53 | 93.18 |
| *PK7* | *HU04G00494.1* | 37.08 | 3.89 | 2.86 | 10.51 | 4.44 | 2.76 |
| *PK8* | *HU05G00291.1* | 40.26 | 6.56 | 11.17 | 23.65 | 13.01 | 16.2 |
| *PK9* | *HU09G00466.1* | 84.95 | 57.56 | 39.68 | 38.22 | 40.14 | 39.74 |
| *PK10* | *HU09G00634.1* | 49.96 | 60.01 | 66.39 | 49.06 | 35.65 | 41.81 |
| *PK11* | *HU09G01922.1* | 29.1 | 9.26 | 5.64 | 18.85 | 17.56 | 14.8 |
| Succinyl-CoA synthetase | | | | | | | |
| *SCS1* | *HU02G02193.1* | 51.55 | 36.82 | 36.58 | 70.71 | 71.57 | 60.29 |
| *SCS2* | *HU03G02309.1* | 61.28 | 32.26 | 40.91 | 60.23 | 47.14 | 44.04 |
| *SCS3* | *HU10G01334.1* | 55.71 | 48.42 | 40.46 | 52.02 | 62.17 | 46.27 |
| Succinate dehydrogenase | | | | | | | |
| *SDH1* | *HU01G02379.1* | 6.11 | 8.27 | 13.87 | 6.76 | 5.93 | 5.11 |
| *SDH2* | *HU05G02019.1* | 78.08 | 60.99 | 54.24 | 64.92 | 80.64 | 65.92 |
| *SDH3* | *HU06G00028.1* | 0.93 | 3.96 | 1.22 | 0.18 | 2.25 | 2.71 |

Table S10 Correlation analyses between the main organic acid contents and expression patterns of candidate genes in 'WCHL' and 'YCHL' pitayas.

| *Gene names* | *Gene ID* | Malic acid | Citric acid | Citromalic acid | Oxalic acid | Fumaric acid | Succinic acid |
|---|---|---|---|---|---|---|---|
| *ACO1* | *HU01G01718.1* | 0.672** | 0.35 | -0.433 | -0.560* | -0.023 | 0.222 |
| *ACO2* | *HU01G01740.1* | 0.827** | -0.302 | 0.201 | -0.364 | .572* | -0.001 |
| *ACO3* | *HU08G00194.1* | 0.082 | 0.331 | -0.319 | -0.442 | -0.272 | 0.198 |
| *CMS1* | *HU01G02605.1* | - | - | 0.948** | - | - | - |
| *CMS2* | *HU06G01828.1* | - | - | 0.327 | - | - | - |
| *CMS3* | *HU08G00711.1* | - | - | 0.924** | - | - | - |
| *CS1* | *HU01G00929.1* | -0.02 | -0.094 | - | -0.139 | 0.477* | 0.295 |
| *CS2* | *HU02G00183.1* | -0.480* | -0.368 | - | 0.302 | 0.476* | -0.007 |
| *CS4* | *HU03G01582.1* | -0.118 | -0.376 | - | 0.156 | 0.702** | 0.174 |
| *CS6* | *HU06G02202.1* | -0.459 | -0.431 | - | 0.28 | 0.495* | 0.077 |
| *CS10* | *HU08G00662.1* | -0.307 | 0.14 | - | -0.114 | 0.238 | 0.517* |
| *CS12* | *HU10G00190.1* | -0.507* | -0.449 | - | 0.316 | 0.480* | 0.048 |
| *DLST1* | *HU01G01093.1* | 0.312 | -0.626** | - | 0.146 | 0.631** | -0.286 |
| *DLST2* | *HU08G00217.1* | -0.085 | 0.043 | - | 0.25 | -0.267 | -0.278 |
| *DLST3* | *HU10G01974.1* | 0.611** | 0.143 | - | -0.252 | 0.025 | 0.029 |
| *Eno4* | *HU09G00620.1* | 0.039 | 0.245 | 0.363 | -0.33 | 0.206 | 0.454 |
| *Eno5* | *HU09G00621.1* | -0.471* | -0.424 | 0.838** | 0.316 | 0.458 | 0.071 |
| *FUM1* | *HU02G00881.1* | -0.745** | 0.504* | - | -0.032 | -0.297 | 0.524* |
| *FUM2* | *HU06G01498.1* | 0.252 | -0.367 | - | -0.015 | 0.273 | -0.378 |
| *MDH1* | *HU06G02301.1* | -0.762** | 0.35 | - | 0.053 | -0.195 | 0.487* |
| *OGDH2* | *HU01G02567.1* | -0.001 | 0.522* | - | -0.131 | -0.329 | -0.007 |
| *OGDH3* | *HU04G02070.1* | 0.844** | -0.577* | - | -0.112 | 0.689** | -0.266 |
| *OGDH6* | *HU06G01674.1* | -0.112 | 0.451 | - | -0.212 | -0.418 | 0.024 |
| *PDH3* | *HU07G00191.1* | -0.343 | -0.547* | 0.892** | 0.314 | 0.577* | -0.037 |

| | | | | | | | |
|---|---|---|---|---|---|---|---|
| *PDH4* | *HU10G00282.1* | -0.25 | -0.447 | 0.938** | 0.192 | 0.654** | 0.108 |
| *PEPC3* | *HU01G00987.1* | -0.102 | 0.804** | -0.431 | -0.468 | -0.499* | 0.375 |
| *PEPC4* | *HU01G00988.1* | -0.143 | 0.768** | -0.425 | -0.415 | -0.532* | 0.31 |
| *PEPC7* | *HU01G00991.1* | -0.193 | -0.011 | -0.195 | -0.109 | -0.184 | 0.136 |
| *PEPC13* | *HU03G01008.1* | 0.529* | -.661** | 0.234 | 0.147 | 0.45 | -0.28 |
| *PEPC14* | *HU03G01874.1* | -0.482* | -0.363 | 0.899** | 0.241 | 0.489* | 0.055 |
| *PEPC16* | *HU08G01056.1* | -0.338 | -0.21 | 0.067 | 0.198 | -0.008 | 0.152 |
| *PEPCK* | *HU10G00784.1* | 0.803** | -0.288 | -0.044 | -0.131 | 0.346 | -0.226 |
| *PGK4* | *HU06G01772.1* | -0.374 | -0.004 | 0.529* | 0.037 | 0.202 | 0.351 |
| *PK6* | *HU03G02352.1* | -0.331 | -0.258 | 0.815** | 0.171 | 0.518* | 0.305 |
| *PK9* | *HU09G00466.1* | -0.092 | -0.549* | 0.937** | 0.126 | 0.706** | -0.014 |
| *SCS1* | *HU02G02193.1* | -0.608** | 0.607** | - | -0.255 | -0.517* | 0.546* |
| *SCS2* | *HU03G02309.1* | -0.741** | -0.049 | - | 0.367 | -0.096 | 0.072 |
| *SCS3* | *HU10G01334.1* | -0.24 | 0.423 | - | -0.378 | 0.104 | 0.746** |
| *SDH1* | *HU01G02379.1* | 0.314 | -0.324 | - | 0.418 | -0.037 | -0.640** |
| *SDH2* | *HU05G02019.1* | -0.357 | 0.325 | - | -0.337 | 0.07 | 0.539* |
| *SDH3* | *HU06G00028.1* | 0.536* | 0.243 | - | -0.272 | 0.056 | 0.095 |
